# The Generation of Genetically Engineered Human Induced Pluripotent Stem Cells Overexpressing IFN-β for Future Experimental and Clinically Oriented Studies

**DOI:** 10.3390/ijms252212456

**Published:** 2024-11-20

**Authors:** Olga Sheveleva, Elena Protasova, Elena Grigor’eva, Nina Butorina, Valeriia Kuziaeva, Daniil Antonov, Victoria Melnikova, Sergey Medvedev, Irina Lyadova

**Affiliations:** 1 Laboratory of Cellular and Molecular Basis of Histogenesis, Koltzov Institute of Developmental Biology of the Russian Academy of Sciences, Moscow 119334, Russia; elenaprotasov@gmail.com (E.P.); nnbut@mail.ru (N.B.); kuzyaeva.valeriya@mail.ru (V.K.); antonovdanya54@gmail.com (D.A.); 2 Laboratory of Developmental Epigenetics, Institute of Cytology and Genetics, Siberian Branch of Russian Academy of Sciences, Novosibirsk 630090, Russia; evlena@bionet.nsc.ru (E.G.); medvedev@bionet.nsc.ru (S.M.); 3 Laboratory of Comparative Developmental Physiology, Koltzov Institute of Developmental Biology of the Russian Academy of Sciences, Moscow 119334, Russia; v_melnikova@mail.ru

**Keywords:** induced pluripotent stem cells, interferon-beta (IFN-β), iPSC ectoderm differentiation, embryoid bodies, PAX6, LHX2

## Abstract

Induced pluripotent stem cells (iPSCs) can be generated from various adult cells, genetically modified and differentiated into diverse cell populations. Type I interferons (IFN-Is) have multiple immunotherapeutic applications; however, their systemic administration can lead to severe adverse outcomes. One way of overcoming the limitation is to introduce cells able to enter the site of pathology and to produce IFN-Is locally. As a first step towards the generation of such cells, here, we aimed to generate human iPSCs overexpressing interferon-beta (IFNB, IFNB-iPSCs). IFNB-iPSCs were obtained by CRISPR/Cas9 editing of the previously generated iPSC line K7-4Lf. IFNB-iPSCs overexpressed IFNB RNA and produced a functionally active IFN-β. The cells displayed typical iPSC morphology and expressed pluripotency markers. Following spontaneous differentiation, IFNB-iPSCs formed embryoid bodies and upregulated endoderm, mesoderm, and some ectoderm markers. However, an upregulation of key neuroectoderm markers, *PAX6* and *LHX2*, was compromised. A negative effect of IFN-β on iPSC neuroectoderm differentiation was confirmed in parental iPSCs differentiated in the presence of a recombinant IFN-β. The study describes new IFN-β-producing iPSC lines suitable for the generation of various types of IFN-β-producing cells for future experimental and clinical applications, and it unravels an inhibitory effect of IFN-β on stem cell neuroectoderm differentiation.

## 1. Introduction

Induced pluripotent stem cells (iPSCs) can be derived from a variety of cell sources; they have an almost unlimited expansion potential, are suitable for genetic engineering, and can be differentiated into diverse types of somatic cells [1,2]. The combination of these properties makes iPSCs a promising tool for treating currently incurable diseases, especially if the cells are modified in a desired way.

Type I interferons (IFN-Is) are pleiotropic cytokines well known for their host-protective antiviral effect. The underlying mechanisms include the ligation of IFN-I by an interferon I receptor (IFNAR) and the down-stream induction of interferon regulatory factors (IRFs) and interferon-stimulated genes (ISGs) [3,4]. ISGs mediate antiviral control by exhibiting direct antiviral activity as well as by modulating the activation, effector function, and trafficking of innate and adaptive immune cells [5,6,7,8].

The role of IFN-Is in nonviral infections is still not entirely clear to researchers. In this context, both protective and detrimental properties of IFN-Is have been reported, with the predominance of the latter ones [9,10,11,12]. The reason seems to be that ISGs lack direct antibacterial activity, and the effect of IFN-I is largely mediated through a modulation of immune cell activities. As a result, the outcomes of IFN-I response depend on multiple factors (i.e., pathogen biology, the route and the stage of infection, the level of pathogen-induced IFN-I secretion, etc.) that are difficult to take into account and to systemize across different studies [9,10,11,12]. Noteworthily, most of the current knowledge on the role of IFN-I during bacterial infections comes from studies performed on mice or on cells with a complete lack of IFN-I signaling [13,14,15,16,17,18]. The approach does not answer the question of how the levels of IFN-I production affect cell antibacterial properties. In this regard, it would be helpful to have an in vitro model that would allow us to measure the antibacterial responses of a given immune cell population intrinsically producing IFN-I at elevated levels.

Besides antiviral and anti-/pro-bacterial activities, type I IFNs also exhibit anti-tumor activity [19,20,21,22,23,24,25]. It has been suggested that IFN-Is may enhance the effect of immune checkpoint therapy, and clinical trials exploring the combined effect of immune checkpoint and IFN-I therapies are currently in progress [26,27]. However, the systemic administration of IFN-I proteins may induce severe adverse outcomes, including hepatotoxicity, leukopenia, severe depression, immune suppression, and others [28,29]. In this respect, it is promising to have technology for generating cells able to penetrate tumors, to express type I IFNs, and to release them locally [30,31,32,33].

In the host, type I IFNs are produced by a variety of cell types, including macrophages [34,35]. Macrophages are directly involved in antiviral, antibacterial, and anti-tumor responses. They have a high capacity to migrate to inflammatory sites and to penetrate into pathologically modified tissues, including infected and neoplastic ones [36,37,38,39]. Taking into account these considerations, we decided to generate macrophages with an intrinsically increased expression of *IFNB* and to explore their antibacterial and anti-tumor properties. To generate IFN-I-overexpressing macrophages, we chose to use a model of iPSC-derived human macrophages well established in the laboratory [40,41]. The advantages of the model are that it allows us (i) to generate macrophages overexpressing a target gene [41]; (ii) to develop macrophages being as close to “natural” tissue macrophages as possible [42,43], and (iii) to obtain macrophages in high quantities [44,45]. As a first step towards the generation of *IFNB*-overexpressing macrophages, we set the task to generate iPSC cell lines with a constitutive overexpression of the *IFNB* gene (IFNB-iPSCs).

Here, we describe the generation and the basic characteristics of IFNB-iPSC lines. We also present evidence that IFN-β, either produced endogenously or being added exogenously, disrupts the ectodermal differentiation of pluripotent stem cells. The results extend our knowledge of IFN-I biological activities and create a model for studying the effect of IFN-β on human embryonic development and on the functionality of various cells that can be generated from *IFNB*-overexpressing iPSCs created in our study.

## 2. Results

### 2.1. The Generation of iPSC Lines Overexpressing IFNB

**Figure 1:**
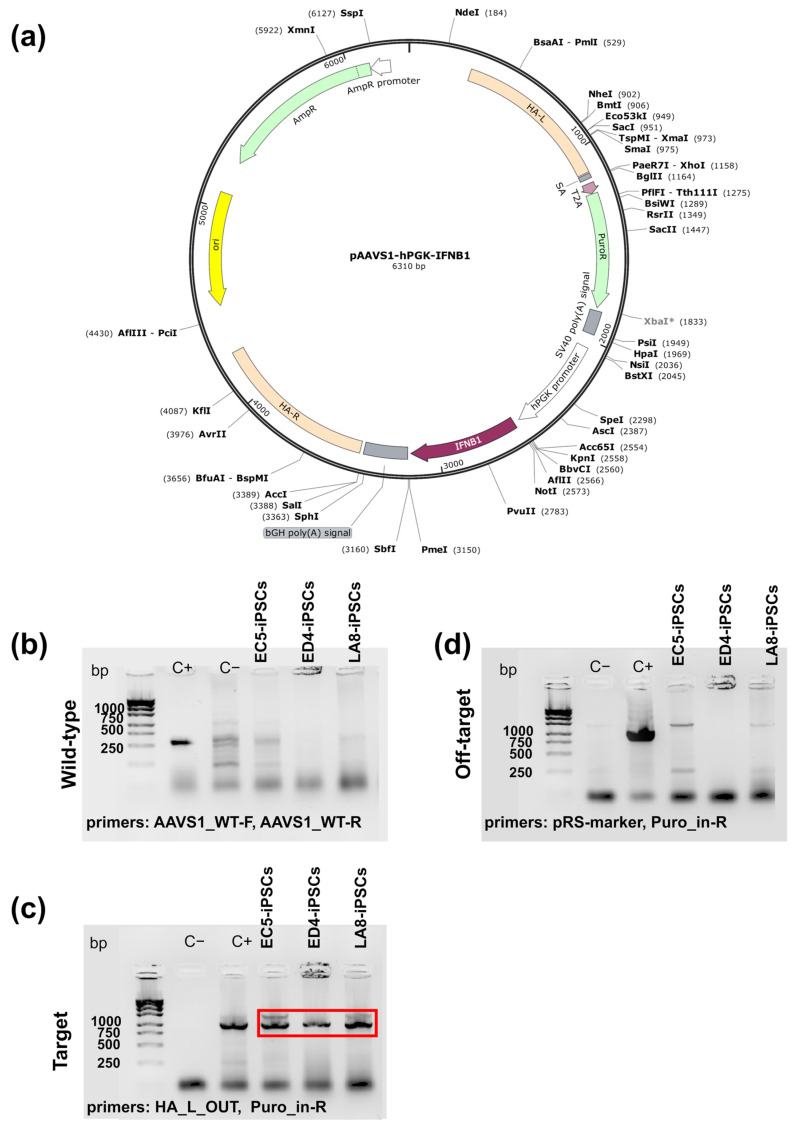
The generation of IFNB-overexpressing human iPSC lines. The ORF of the human IFNB gene was inserted into the AAVS1 locus of parental iPSC line K7-4Lf using the CRISPR/Cas9 technology. In brief, K7-iPSCs were transfected with pAAVS1-hPGK-IFNB1 and sgRNA-Cas9 plasmids; successfully transfected clones were selected in a puromycin-containing medium; the selected clones were expanded and screened for the presence of on-target and the absence of off-target inserts. (**a**) pAAVS1-hPGK-IFNB1 plasmid scheme; (**b**–**d**) representative gels showing the results of iPSC screening for on-target and off-target inserts; (**b**) representative gel showing the results of iPSC screening for the presence of unmodified wild-type AAVS loci (WT AAVS1). C+, positive control, parental K7-iPSCs containing unmodified AAVS1 loci; C−, negative control, a previously generated iPSC line with a proven correct insert of the roGFP2-Orp1 transgene into the AAVS1 locus [47]; (**c**) representative gel showing the results of on-target screening in edited iPSCs. C+, iPSC line with a proven correct insert of the roGFP2-Orp1 transgene into the AAVS1 locus [47]. C−, parental K7-iPSC line; (**d**) representative gel showing the results of off-target screening in edited iPSCs. C+, pAAVS1-hPGK-IFNB1 plasmid; C−, iPSC line with a proven correct insert into the AAVS1 locus. iPSCs, induced pluripotent stem cells; K7-iPSCs, parental K7-4Lf iPSC line.

To generate IFNB-iPSCs, we used iPSC line K7-4Lf (alternative name: ICGi022-A [46]; hereafter referred to as K7-iPSCs) previously generated from human blood mononuclear cells of a healthy woman. K7-iPSCs were electroporated with plasmids carrying (i) the components of the CRISPR/Cas9 system (sgRNA and SpCas9) and (ii) the hPGK promoter-driven ORF of the human IFNB gene for integration into the human AAVS1 locus (Figure 1a). After puromycin selection and the examination of the newly generated clones for the presence of on-target and off-target inserts (Figure 1b–d), 22 cell clones were obtained. Of them, 13 clones had correct insertions into both AAVS1 loci (homozygous clones) and 9 clones had correct insertions into a single AAVS1 locus (heterozygous clones). For a further analysis, two homozygous clones (LC8 and LA8) and one heterozygous clone (LE4) were selected and expanded (Supplementary Figure S1).

### 2.2. IFNB-iPSCs Display Morphological and Phenotypic Characteristics of Pluripotent Stem Cells

**Figure 2:**
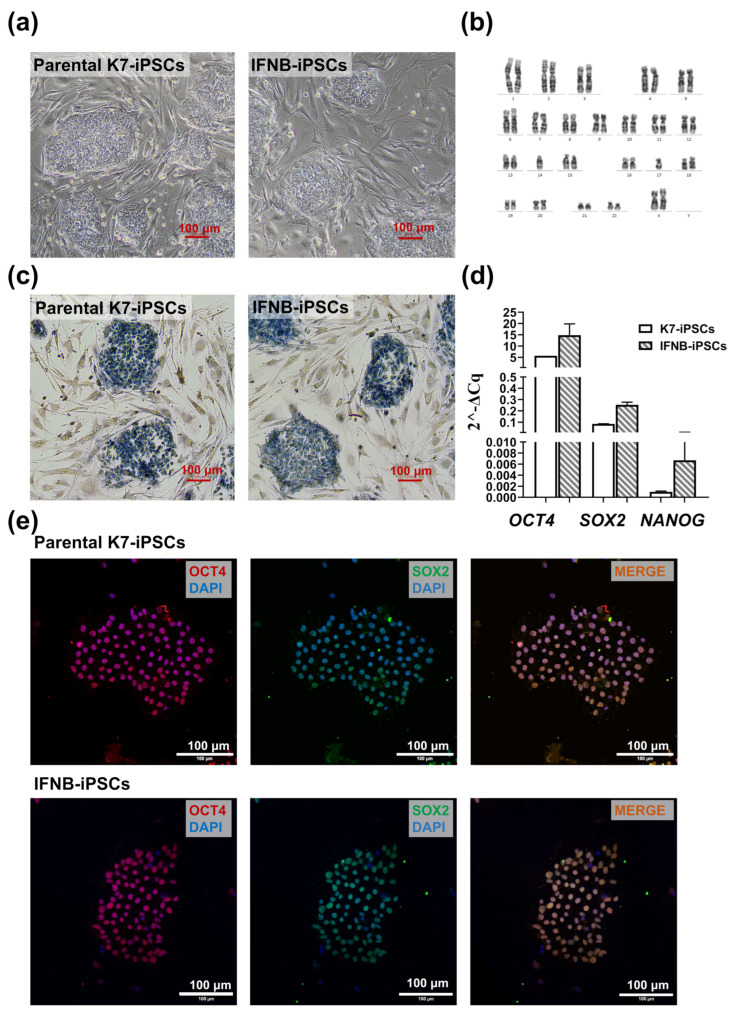
IFNB-iPSC lines display the morphological and phenotypic characteristics of pluripotent cells and a normal karyotype. At least three IFNB-iPSC lines, LA8-iPSCs, LC8-iPSCs, and LE4-iPSCs, were expanded and examined in each of the indicated assays. The results obtained using IFNB-iPSC line LA8 are shown as representative. (**a**) Light microscopy of LA8-iPSCs and parental K7-iPSCs growing on mouse embryonic fibroblast feeder cells. Note the similar morphology of IFNB-iPSCs and K7-iPSCs. Phase contrast. 10× magnification. (**b**) The karyogram of LA8-iPSCs. (**c**) Positive immunohistochemical staining of LA8-iPSCs for alkaline phosphatase. K7-iPSCs were used as a positive control. (**d**) The expression of pluripotency markers *OCT4*, *SOX2*, and *NANOG* by LA8-iPSCs (RT-qPCR; representative experiment, n = 3; median with 95% CI). (**e**) The expression of OCT4 (red) and SOX2 (green) pluripotency proteins in LA8-iPSCs. Note similar patterns of protein expression in IFNB-iPSCs and the parental K7-iPSCs. Immunofluorescence staining, confocal microscopy (Zeiss LSM 880 microscope; Carl Zeiss, Jena, Germany). Nuclei were stained with DAPI (blue). In all images, the scale bar is 100 µm. IFNB-iPSCs, iPSCs with a constitutive overexpression of the IFNB gene; LA8-iPSCs, IFNB-iPSC line LA8; LC8-iPSCs, IFNB-iPSC line LC8; LE4-iPSCs, IFNB-iPSC line LE4.

The morphological analysis showed that all selected IFNB-iPSC lines (i.e., LA8-, LC8-, and LE4-iPSC lines) had a morphology typical for iPSCs; i.e., they grew as flat colonies without inter-cell spaces, they had large nuclei with visible nucleoli, and they formed clear borders with the feeder cells (Figure 2a; Supplementary Figure S2). *IFNB* overexpression did not affect cell viability: the percentages of live cells detected by cell staining with trypan blue or with a fixable viability dye were similarly high in IFNB-iPSCs and K7-iPSCs (Supplementary Figure S3). The growth potential of the IFN-iPSCs was similar to that of the parental K7-iPSCs: being passaged with a similar density, the cells reached a 60–70% confluence within a similar time frame. In the karyotypic analysis, the lines displayed a normal 46, XX karyotype identical to that of the parental K7-iPSC line (Figure 2b).

To further examine the pluripotency of IFNB-iPSC lines, we determined the activity of alkaline phosphatase and evaluated the expression of canonical pluripotency markers using immunofluorescence and RT-qPCR approaches. All the tested lines were positive for alkaline phosphatase activity with no evident differences observed between them and the parental K7-iPSC line (Figure 2c). In RT-PCR, IFNB-iPSCs expressed octamer-binding transcription factor 4 (*OCT4*), SRY-Box transcription factor 2 (*SOX2*), and *NANOG* at levels similar to or even higher than those displayed by K7-iPSCs (Figure 2d). Immunostaining confirmed the expression of OCT4 and SOX2 at the protein level (Figure 2e).

The pluripotency phenotype of the newly generated IFNB-iPSCs was stable: following prolonged cultures, the cells retained their morphological characteristics, self-renewal capacities, and pluripotency marker expression (tested at passages 28–30).

### 2.3. IFNB-iPSCs Overexpress Functional IFN-β

**Figure 3:**
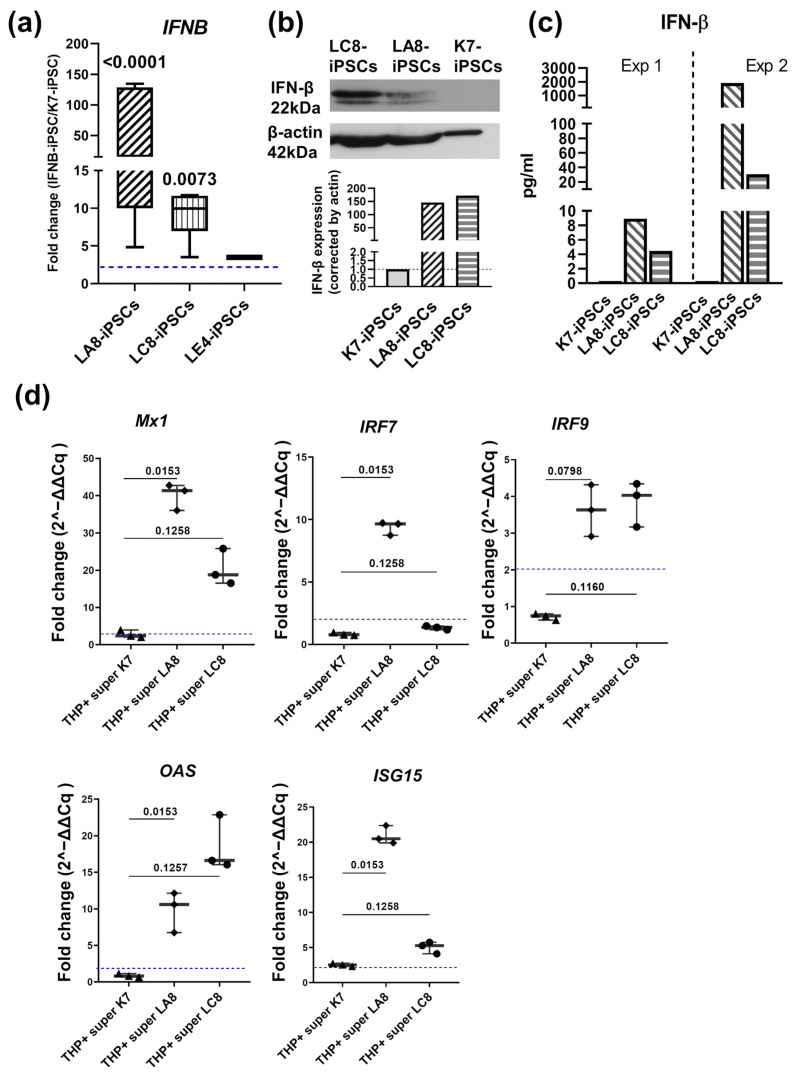
IFNB-iPSCs express functional IFN-β. IFNB-iPSCs and K7-iPSCs were cultured in parallel and used for RNA isolation; the preparation of cell extracts; and the collection of cell culture supernatants. (**a**) IFNB-iPSCs display an increased expression of *IFNB* as compared with the parental K7-iPSC line. RNAs isolated from IFNB-iPSCs and K7-iPSCs were subjected to RT-PCR; *IFNB* expression values were normalized relative to *GAPDH*, and fold changes were calculated as relative IFNB mRNA levels in IFNB-iPSCs relative to K7-iPSCs (2^−∆∆Cq^). Dotted line, fold change = 2 (significance threshold). Data are shown as boxes and whiskers with minimal and maximal values (K7-iPSCs, LA8-iPSCs, and LC8-iPSCs, summarized results of at least 3 independent experiments; LE4-iPSCs, one experiment, technical replicates). Note that LA8-iPSCs and LC8-iPSCs bear a homozygous *IFNB* insertion, whereas LE4-iPSCs are heterozygous. The significance of the differences was determined using the two-stage linear step-up procedure of Benjamini, Krieger, and Yekutieli. (**b**,**c**) IFNB-iPSCs express IFN-β at the protein level. (**b**) IFNB-iPSCs and K7-iPSCs were pelleted, frozen, and analyzed in a Western blot. The graph shows the relative densitometric values of IFN-β normalized to β-actin. (**c**) The supernatants were collected from IFNB-iPSCs and K7-iPSCs and analyzed using ELISA (the results of two independent experiments; in each of them, K7-iPSCs, LA8-iPSCs, and LC8-iPSCs were cultured in parallel and independently of cells of another experiment). (**d**) The supernatants of IFNB-iPSCs exhibit IFN-β-like functional activity. The supernatants were obtained from IFNB-iPSC and K7-iPSC cultures and were added to THP-1 macrophage-like cells pre-activated with PMA. Control THP-1 cells were cultured in the absence of iPSC supernatants. THP-1 RNA was isolated from all cultures, and the expressions of ISGs were analyzed in RT-qPCR using *RPL27* as a housekeeping gene. Data are shown as boxes and whiskers with minimal and maximal values and individual points. The representative results of one out of two experiments are presented. Numbers on the graphs show the FDRs (Benjamini, Krieger, and Yekutieli correction for multiple comparisons). FDRs < 0.05 were considered as significant; for the comparison of IFNB-iPSCs and K7-iPSCs, FDRs > 0.05 are also shown. The differences between LA8-iPSCs and LC8-iPSCs were insignificant. PMA, phorbol 12-myristate-13-acetate.

To check *IFNB* expression in the generated IFNB-iPSC lines, we compared the levels of *IFNB* RNA in IFNB-iPSCs and in the parental K7-iPSCs (Figure 3a). Homozygous IFNB-iPSC lines LA8 and LC8 expressed *IFNB* at significantly higher levels, as compared with K7-iPSCs (fold increase in LA8- and LC8-iPSCs as compared with K7-iPSCs, 51.8 and 27.8, respectively; Figure 3a). In the heterozygous LE4-iPSCs, the level of *IFNB* expression was not significantly elevated (FDR > 0.1). Therefore, our further analyses were performed using only the two homozygous IFNB-iPSC lines, LA8 and LC8. Among them, the LA8 cell line exhibited the highest level of *IFNB* expression (Figure 3a). An increased expression of *IFNB* persisted until at least passage 28 (the last observation point), indicating that the genetic modification introduced to IFNB-iPSCs was stable.

To check whether IFNB-iPSCs expressed IFN-β at the protein level, we performed Western blotting and ELISA. In both tests, we did not detect IFN-β production in K7-iPSCs, but the protein was readily identified in IFNB-iPSCs (Figure 3b,c). Western blot densitometry showed somewhat higher levels of IFN-β in LC8-iPSCs as compared with LA8-iPSCs, which contrasted with the results obtained at the mRNA level. In ELISA, the levels of IFN-β secretion by LA8-iPSCs were much higher than those in LC8-iPSCs. The latter was well in line with the results obtained at the mRNA level and suggested a more efficient secretion of IFN-β by LA8-iPSCs (i.e., a lower retention of IFN-β in LA8-iPSCs). Some fluctuations in the levels of IFNB/IFN-β expression by each IFNB-iPSC line could also not be excluded. Regardless of the explanation for the results, IFNB-iPSCs produced more IFN-β than parental K7-iPSCs, and it was important to determine whether the secreted IFN-β was functional.

To carry out this, we analyzed whether the supernatants of IFNB-iPSC cultures could induce the expression of ISGs in human macrophages. We collected the supernatants from the cultures of LA8 and LC8 IFNB-iPSCs, as well as from the control K7-iPSCs, and added them to PMA-activated THP-1 macrophage-like cells. Twenty-four hours later, we isolated THP-1 RNA and analyzed the expression of the following ISGs by RT-PCR: mycovirus resistance 1 (*MX1*), 2′-5′-oligoadenylate synthetase 1 (*OAS*), interferon-stimulated gene 15 (*ISG15*), interferon regulatory factors 7 (*IRF7*), and interferon regulatory factors 9 (*IRF9*) (Figure 3d). THP-1 macrophages cultured in the absence of iPSC-derived supernatants served as controls. We registered a significant upregulation of ISG expression in THP-1 cells treated with the supernatants derived from IFNB-iPSCs but not from K7-iPSCs (fold changes relative to untreated THP1, >2). We also noted that LA8 supernatants induced a more pronounced effect as compared with the supernatants from LC8 cells. This corresponds well to a higher-level secretion of IFN-β by the former IFNB-iPSC line (Figure 3c). Although we did not directly explore whether the effect of IFN-β was dose-dependent and iPSC lines may secrete a number of factors besides IFN-β, this interpretation is well in line with published data showing that IFN-I induces the upregulation of ISGs in a dose-dependent manner [48,49].

We concluded that both IFNB-iPSC lines exhibited a stable expression of *IFNB* and produced a functionally active IFN-β protein.

### 2.4. IFNB-iPSCs Exhibit a Capacity to Spontaneously Differentiate into Endodermal and Mesodermal Directions But Their Ectodermal Differentiation Is Disrupted

One of the characteristic features of iPSCs is their capacity to spontaneously form EBs and differentiate into the three germ layers [50,51,52]. To further characterize IFNB-iPSC lines, we cultured them in low-adhesive conditions, which are known to stimulate the formation of EBs [53]. IFNB-iPSC lines LA8 and LC8 were cultured in parallel with K7-iPSCs. On days 20–21, EBs derived from IFNB-iPSCs (IFNB-EBs) and K7-iPSCs (K7-EBs) were analyzed for the expression of endoderm-, mesoderm-, and ectoderm-associated markers using immunofluorescence and RT-PCR approaches.

**Figure 4:**
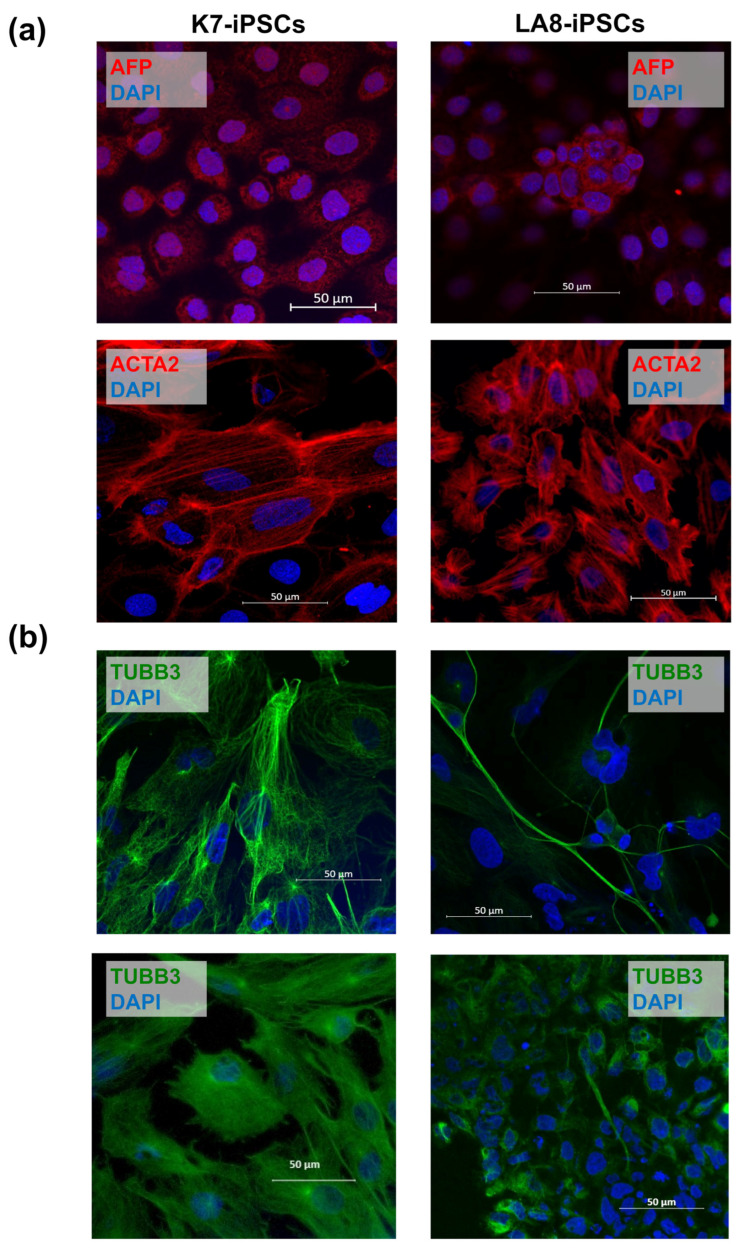
The immunofluorescence analysis reveals the expression of endoderm-, mesoderm-, and ectoderm-associated proteins in embryonic bodies spontaneously differentiated from IFNB-iPSCs. IFNB-iPSC lines LA8 and LC8 and parental K7-iPSCs were cultured in low-adhesive conditions to stimulate the formation of embryoid bodies (EBs). On day 15, EBs were transferred to Matrigel-coated coverslips to induce the formation of cell monolayers; 6 days later, the cells were stained with antibodies specific to endoderm, mesoderm, and ectoderm markers and analyzed by confocal microscopy (Zeiss LSM 880 (Carl Zeiss, Jena, Germany)). Scale bar: 50 µm. (**a**) Immunofluorescence staining of spontaneously differentiated EBs for germ layer markers AFP (endoderm) and ACTA2 (mesoderm). The results obtained using IFNB-iPSC line LA8 and parental K7-iPSCs are shown. Similar results were obtained using IFNB-iPSC line LC8 (Supplementary Figure S4). (**b**) Immunofluorescence staining of spontaneously differentiated EBs for germ layer marker TUBB3 (ectoderm). Two different fields of view for IFNB-EBs (line LA8 as a representative) and K7-EBs are shown. ACTA2, actin alpha 2; EBs, embryoid bodies; TUBB3, tubulin beta-3.

In the immunofluorescence analysis, we evaluated the expression of alpha-fetoprotein (AFP, the marker of the endoderm [54,55]), actin alpha 2 (ACTA2, the marker of the mesoderm [55,56,57]), and tubulin beta-3 Class III (TUBBIII, an early marker of the ectoderm [55,56,58,59]). AFP and ACTA2 were expressed in all analyzed EBs with no evident differences between IFNB-EBs and K7-EBs (Figure 4a). The expression of TUBB3 appeared as characteristic fibers, and it was also detected in the cell cytoplasm. The protein could be identified in all tested EBs; however, quantitative inter-sample comparisons were difficult to perform since TUBB3 expression manifested itself with different intensities, lengths, and shapes in different fields of view even within the same sample (Figure 4b).

**Figure 5:**
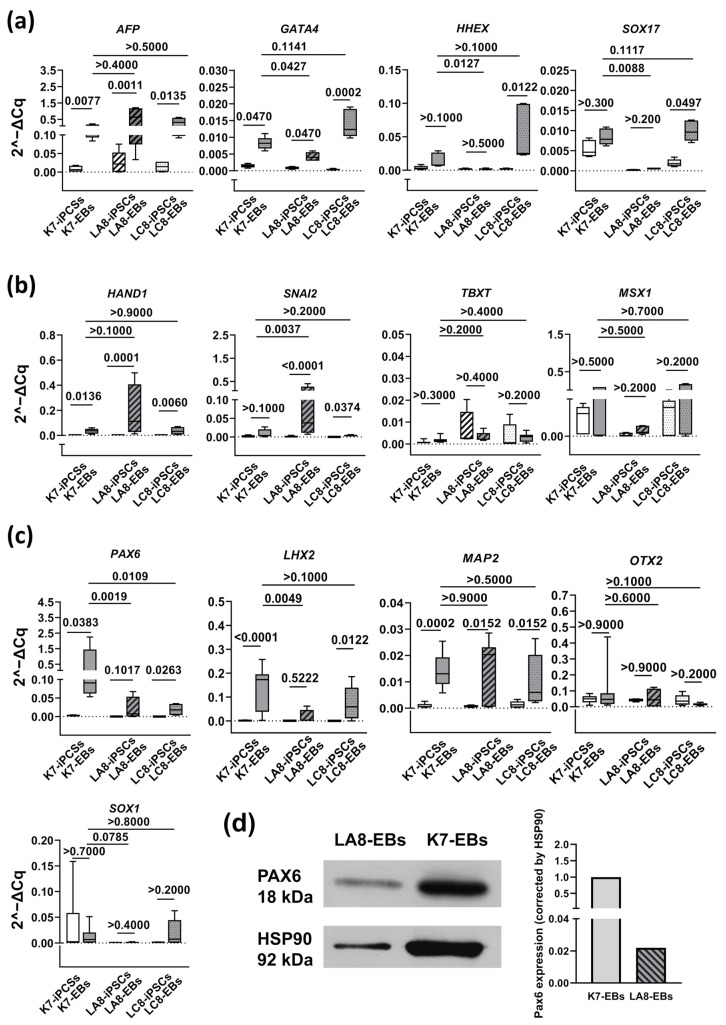
A disrupted expression of ectoderm-associated genes in embryonic bodies spontaneously differentiated from IFNB-iPSCs. IFNB-iPSC lines LA8 and LC8 and parental K7-iPSCs were cultured in low-adhesive conditions to stimulate the formation of embryoid bodies (EBs). On day 20, RNA was isolated and gene expressions were analyzed using RT-PCR. Whole-cell lysates of EBs were also analyzed by a Western blot. (**a**–**c**) Relative expressions of endoderm- (**a**), mesoderm- (**b**), and ectoderm- (**c**) associated markers in iPSCs and 20-day EBs. Data are shown as boxes and whiskers with minimal and maximal values (summarized results of at least 2 independent experiments). The significance of the differences was determined using the two-stage linear step-up procedure of Benjamini, Krieger, and Yekutieli. Figures indicate the FDRs for the main comparisons, irrespectively of their significance (i.e., inter-line comparisons on differentiation day 20 and intra-line comparisons between iPSCs and 20-day EBs). (**d**) The Western blot analysis of PAX6 protein in IFNB-EBs and K7-EBs (one experiment). The graph shows the relative densitometric values of PAX6 normalized to HSP90.

When analyzing the differentiation potential of IFNB-iPSCs using RT-PCR, we included in the analysis at least four markers for each of the analyzed germ layers. The list of endoderm markers included AFP, GATA-binding protein 4 (*GATA4*), haematopoietically expressed homeobox (*HHEX*), and SRY-Box transcription factor 17 (*SOX17*) [58,60,61,62,63] (Figure 5a). Following the differentiation of iPSCs into EBs, in each of the analyzed cell lines, at least two of the four analyzed endoderm genes were significantly (FDR < 0.05) upregulated (lines K7-4Lf and LA8: *AFP* and *GATA4*; line LC8: *AFP*, *GATA4*, *HHEX*, and *SOX17*). The comparison of the levels of gene expression in IFNB-EBs and K7-EBs on differentiation day 20 revealed a lower expression of *GATA4*, *HHEX*, and *SOX17* in LA8-EBs, but no significant decline in the expression of the analyzed genes in LC8-EBs. Moreover, there was a tendency towards a higher expression of *GATA4* in LC8-EBs as compared with K7-EBs (FDR, 0.1141, *p* = 0.0317 if applying the Mann–Whitney test). We concluded that although there were some differences in endoderm gene expression between IFNB-EBs and K7-EBs, the changes did not have a stable pattern.

To evaluate mesoderm differentiation, we analyzed the expression of heart and neural crest-derived transcript 1 (*HAND1*), snail family transcriptional repressor 2 (*SNAI2*), T-box transcription factor T (*TBXT*), and *MSX1* [58,60,61,62]. Following the differentiation of all iPSC lines, *HAND1* and *SNAI2* were significantly upregulated (FDR < 0.04; Figure 5b). There was also a tendency towards an upregulation of the *TBXT* gene in K7-EBs (FDR > 0.1; *p* = 0.0401, Mann–Whitney test). The comparison of the expression of the genes in IFNB-EBs and K7-EBs did not reveal any significant differences except for a higher expression of *SNAI2* in LA8-EBs. We interpreted these results as a lack of a stable influence of *IFNB* overexpression on mesoderm differentiation in our model.

To examine ectodermal differentiation, we assessed the expression of the paired box 6 (*PAX6*), LIM homeobox 2 (*LHX2*), microtubule-associated protein 2 (*MAP2*), orthodenticle homeobox 2 (*OTX2*), and SRY-Box transcription factor 1 (*SOX1*) genes, which in other studies were used as and referred to as markers of cell ectoderm and/or neural differentiation [58,61,62,63,64]. Following the differentiation of K7-iPSCs, the expression of the *PAX6*, *LHX2*, and *MAP2* genes increased significantly, indicating an ongoing ectodermal differentiation in K7-EBs (FDR for the comparison of the three genes in K7-EBs versus K7-iPSCs, 0.0383, <0.0001 and 0.0002, respectively; Figure 5c). The expression levels of the other two ectoderm-associated genes, *OTX2* and *SOX1*, did not increase and remained at similar levels in K7-iPSCs and K7-EBs (see Discussion for a possible explanation of the results).

Following the differentiation of IFNB-iPSCs, among the five tested ectoderm-associated markers, only the expression of the *MAP2* gene upregulated reproducibly in both tested IFNB-iPSC lines (FDR for the comparison of LA8-EBs and LA8-iPSCs and for the comparison of LC8-EBs and LC8-iPSCs, 0.0152). The *PAX6* and *LHX2* expressions upregulated during the differentiation of LC8-iPSCs (FDR, 0.0263 and 0.0122, respectively), but did not increase following the differentiation of LA8-iPSCs (FDR, 0.1017 and 0.5222 for *PAX6* and *LHX2* genes, respectively). A comparison between the gene expression levels in 20-day IFNB-EBs and 20-day K7-EBs showed that LA8-EBs displayed a significantly diminished expression of *PAX6* and *LHX2* (FDR < 0.005) and a tendency towards a lower expression of *SOX1* (FDR, 0.0785). In LC8-EBs, the expression of *PAX6* was also decreased (FDR, 0.0109). Noteworthily, in LA8-EBs, the suppression of ectoderm marker expression was more intense as compared with LC8-EBs. As we showed above, LA8-iPSCs expressed *IFNB* and secreted IFN-β at higher levels as compared with LC8-iPSCs. This indirectly supported our assumption about a negative effect of *IFN-β* on cell ectoderm differentiation.

PAX6 is a key early neuroectoderm differentiation marker. To check whether *IFNB* overexpression affected its expression at the protein level, we performed a Western blot analysis of spontaneously differentiated LA8-EBs and K7-EBs. We found a 45-fold decrease in PAX6 expression in LA8-EBs as compared with K7-EBs (Figure 5d), which was well in line with our data obtained at the mRNA level.

Theoretically, a diminished expression of some differentiation markers in EBs could result from cell dedifferentiation. To check this possibility, we analyzed the expression of OCT4 pluripotency markers in IFNB-EBs and K7-EBs (Supplementary Figure S5). In the immunofluorescence analysis, OCT4 expression was detected neither in LA8-EBs nor in K7-EBs. In Western blotting, the OCT4 band was clearly detected in LC8-iPSCs, but not in LC8-EBs. The results suggested that the impairment of neuroectoderm gene expression seen in IFNB-EBs was not due to cell dedifferentiation.

### 2.5. Exogenous IFN-β Disrupts iPSC Ectodermal Differentiation

**Figure 6:**
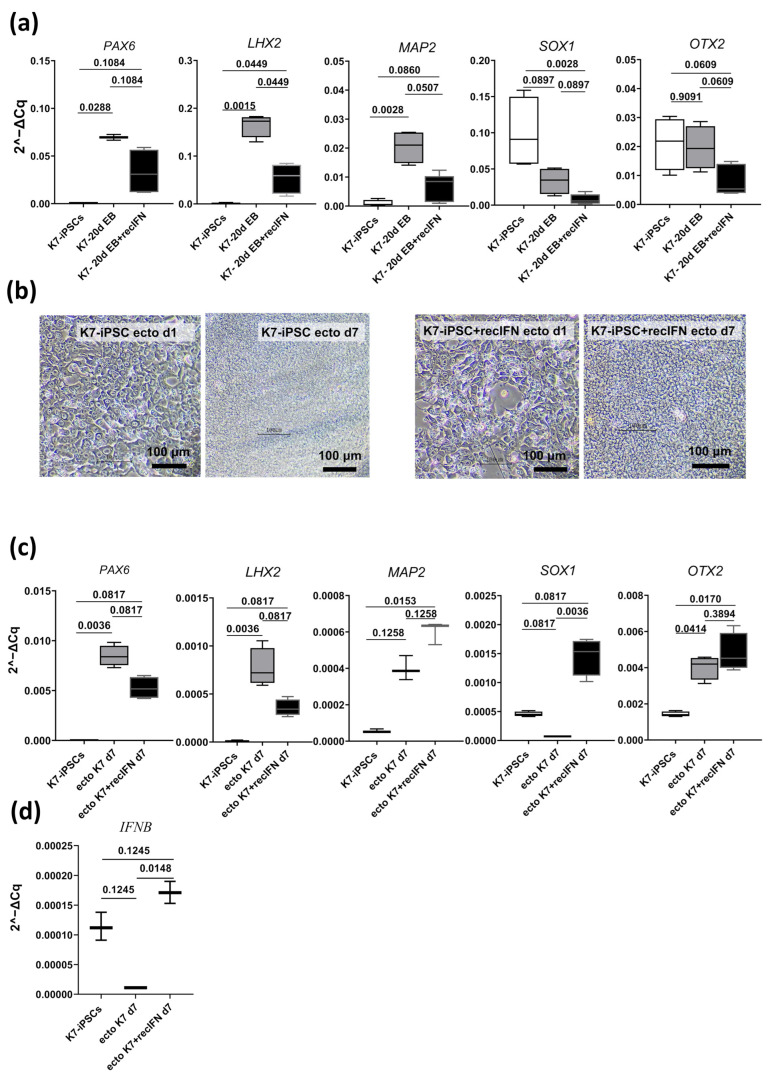
Exogenous IFN-β disrupts the expression of ectoderm-associated genes in differentiating parental K7-iPSCs. K7-iPSCs were subjected to spontaneous (**a**) or directed (**b**–**d**) differentiation in the absence or in the presence of recombinant human IFN-β; at the end of the differentiation, the expression of ectoderm-associated markers was analyzed using RT-PCR (housekeeping gene, *RPL27*) and compared with that observed in original K7-iPSCs. The significance of the differences was determined using the two-stage linear step-up procedure of Benjamini, Krieger, and Yekutieli. (**a**) Changes in the expression of ectoderm-associated genes in spontaneously differentiated EBs. Gene expression was analyzed on day 20. Data are shown as boxes and whiskers with minimal and maximal values. The summarized results of two independent experiments are shown. (**b**) Light microscopy of K7-iPSCs differentiating in the absence or in the presence of IFN-β. Made on differentiation days 1 and 7. (**c**) Changes in the expression of ectoderm-associated genes following the directed differentiation of K7-iPSCs in the STEMdiff™ Trilineage Ectoderm Medium. Gene expression was analyzed on day 7 (as recommended by the manufacturer). Data are shown as boxes and whiskers with minimal and maximal values. The representative results of one out of two experiments are presented. (**d**) Changes in the expression of the *IFNB* gene following the directed differentiation of K7-iPSCs in the STEMdiff™ Trilineage Ectoderm Medium (the representative results of one out of two experiments).

Having found that following the spontaneous differentiation of IFNB-iPSCs, the upregulation of ectoderm differentiation markers is hampered, we then wondered whether exogenous IFN-β would exert a similar effect. To explore the issue, we subjected parental K7-iPSCs to spontaneous differentiation in the presence or in the absence of human recombinant IFN-β, and analyzed the expression of ectoderm-associated markers in 20-day EBs (Figure 6).

In the absence of IFN-β, spontaneous differentiation induced a significant upregulation of the *PAX6*, *LHX2*, and *MAP2* genes (fold increase in K7-EBs as compared with original K7-iPSCs, 139, 1488, and 41; FDR, 0.0288, 0.0015, and 0.0028 for the *PAX6*, *LHX2*, and *MAP2* genes, respectively). This was well in line with our results described in Section 2.4. The addition of IFN-β abrogated the upregulation of *PAX6* (FDR, 0.1084 for the comparison of K7-EBs and K7-iPSCs) and *MAP2* (FDR, 0.0860) and attenuated the expression of *LHX2* (2.5-fold as compared with K7-EBs differentiated in the absence of IFN-β, FDR, 0.0449; Figure 6a). The expression of two other ectoderm-associated genes, *SOX1* and *OTX2*, did not increase in the absence of IFN-β and there was even a tendency towards a downregulation of *SOX1* (2.5-fold as compared with K7-iPSCs; FDR, 0.0897). IFN-β made this suggestive difference significant (FDR for the comparison of K7-EBs with original K7-iPSCs, 0.0028).

To further address the effect of IFN-β on iPSC differentiation in the ectodermal direction, we investigated whether IFN-β would interfere with the directed ectoderm differentiation of K7-iPSCs. For that purpose, we cultured K7-iPSCs in a STEMdiff™ Trilineage Ectoderm Medium in the presence or in the absence of recombinant human IFN-β and analyzed ectoderm marker expression 7 days later (as recommended by the manufacturer; Figure 6b,c). In the absence of IFN-β, the differentiation resulted in a significant upregulation of the two main ectoderm-associated genes, *PAX6* and *LHX2* (FDR, 0.0036); *OTX2* was also upregulated (FDR, 0.0414). The addition of IFN-β abrogated the upregulation of *PAX6* and *LHX2* (FDR for the comparison of *PAX6* and *LHX2* expression on day 7 versus day 0, 0.0817). The expression of other tested genes either was not significantly affected by IFN-β (*MAP2* and *OTX2*) or even increased (*SOX1*, FDR, 0.0036). Noteworthily, the expression of *IFNB* decreased following the differentiation of K7-iPSCs in the absence of exogenous IFN-β, but did not decline if IFN-β was added to the differentiating cells, supporting the existence of inverse relationships between *IFNB* expression and neuroectoderm commitment (Figure 6d).

Altogether, iPSC lines generated in this study showed a stable upregulation of *IFNB* expression and displayed morphological and phenotypic characteristics of iPSCs. Functionally, the iPSC-IFN lines were able to differentiate into endoderm and mesoderm directions, but their capacity for ectoderm differentiation was hampered at least in part, as evident from the disrupted expression of two key neuroectoderm markers, *PAX6* and *LHX2*.

## 3. Discussion

In this study, we report on the generation and the characterization of genetically engineered *IFNB*-expressing human iPSC lines and document the negative effects of IFN-β on pluripotent stem cell ectoderm differentiation.

The generation of human iPSCs overexpressing *IFNB* is of interest due to several reasons. First, iPSCs can be differentiated into various human immune and non-immune cells [65,66,67,68]. This creates a basis for generating various populations of IFN-β-overproducing cells of the same origin and for studying how the prolonged overproduction of the cytokine affects the functionality of different cells in different conditions. This is pertinent as current data on the role of IFN-β in many pathological conditions are contradictory. The data are largely based on the use of the *IFNAR* knockout model [13,14,15,16,17,18], and this approach does not answer the question on how quantitative parameters of IFN-β response affect individual cell populations implicated in disease pathogenesis. Second, the in vitro differentiation of iPSCs into diverse cell populations recapitulates the corresponding branches of cell differentiation during embryonic development [69,70]. IFNB-iPSCs provide an accessible model to explore how these processes go on in the conditions of endogenous IFN-β overexpression. Finally, immune cells overproducing IFN-β hold promise as a cell therapeutic tool. As a proof-of-principle, in a xenogeneic experimental model, human iPSC-derived myeloid cell lines engineered to overexpress *IFNB* or *IFNA* and to maintain their proliferative activity accumulated within neoplastic lesions and inhibited human tumor growth [30,31,32,33].

To be categorized as iPSCs, cells need to meet morphological, molecular, and functional criteria [52]. Three different lines of IFNB-iPSCs examined in our study displayed typical iPSC morphology, and they expressed pluripotency-associated transcriptional factors and markers (*SOX2*, *OCT4*, *NANOG*, alkaline phosphatase). These characteristics were similar between IFNB-iPSCs and the parental K7-iPSC line and they are consistent with IFNB-iPSC pluripotency.

In the functional analysis, we examined two homozygous IFNB-iPSC lines, LA8-iPSCs and LC8-iPSCs. Both were capable of spontaneously forming EBs and upregulating endoderm- and mesoderm-associated genes (such as *AFP*, *GATA4*, *HAND1*, and *SNAI2*) and proteins (AFP; ACTA2). Concerning ectoderm markers, the immunofluorescence analysis detected the expression of TUBB3 in 21-day IFNB-EBs. However, RT-PCR revealed a significant impairment of the expression of ectoderm-associated genes in IFNB-EBs, especially in LA8-EBs, which were derived from the LA8-iPSC line with a higher level of *IFNB* expression (as compared with LC8-iPSCs). Significantly, exogenous IFN-β produced a similar effect on the differentiation of parental K7-iPSCs.

There are several possible explanations for these results. First, IFN-β may directly hamper iPSC properties. Second, IFN-β may act during the process of iPSC ectodermal differentiation. Third, the cytokine may change the cell differentiation dynamic without affecting the overall capacity of IFNB-iPSCs for ectoderm differentiation (i.e., the dysregulation of ectoderm gene expression registered in our study could be due to a delayed dynamic of cell differentiation, but not to a complete impairment of ectoderm formation).

The first assumption follows from the fact that pluripotent cells lack an IFN-I system: iPSCs and embryonic stem cells (ESCs) do not express type I IFNs and they poorly respond to type I IFNs and type I IFN-inducing stimuli, including viral infections, dsRNA, and Poly I:C [28,71,72,73,74]. The reactivity to IFN-I and IFN-I-inducing stimuli is acquired following iPSC/ESC differentiation, starting with the stage of formative pluripotency [75,76]. This indicates that at the stage of cell naïve pluripotency, maintaining an IFN-I system in a suppressed state is biologically important and suggests that the expression of IFN-I may be harmful for iPSCs.

In line with this, the IFN-I system deficiency in pluripotent cells is supported by several mechanisms. Early studies attributed it to a low-level expression of receptors initiating IFN-I responses (i.e., toll-like receptor 3 (TLR3), retinoic acid-inducible gene 1 (RIG-1), Melanoma Differentiation-Associated protein 5 (MDA-5), TLR-4, and others) and to an inactive status of NF-κB [73,75]. Later, reciprocal relationships between cell pluripotency and type I IFNs were attributed to the inhibitory effect of Kruppel-Like Factor 4 (KLF4) and other pluripotency-associated transcriptional factors on the IFN-I system [76,77]. In another recent study, a repression of the IFN-I system in pluripotent cells was linked to the activity of Dicer, a key enzyme responsible for miRNA and siRNA biogenesis [74]. Knockout of Dicer in mouse ESCs restored IFN-I response and simultaneously diminished cell proliferative activity, a characteristic feature of pluripotent cells necessary for the maintenance of their self-renewal. According to Romeike and co-authors [76], the inhibition of the IFN-I system is restricted to the stage of naïve pluripotency; exit from the naïve pluripotency state is accompanied by the induction of formative pluripotency transcriptional factors that induce Irf1; the latter induces the expression of a set of ISGs; i.e., it cancels IFN-I system suppression.

To summarize, a lack of IFN-I response is a general characteristic of pluripotency, and it is actively maintained during the pluripotency by pluripotency-associated transcriptional factors. It is supposed that the biological sense of an IFN-I blockade in pluripotent cells is to maximize cell survival, proliferative activity, and self-renewal and to minimize immunological cytotoxicity at the early stages of embryogenesis [78]. However, to the best of our knowledge, there is no direct evidence of any harmful effect of IFN-I on pluripotent cells. IFNB-iPSCs generated in our study provide a suitable model to address this issue.

In our study, the enforced expression of *IFNB* did not impair IFN-iPSC morphology, viability, or growth capacity, nor did it hamper pluripotency marker expression in IFN-iPSCs. Furthermore, following the exposure to recombinant IFN-β, parental K7-iPSCs (whose compliance with pluripotency criteria had been reported [46] and was additionally confirmed in this study) did not change their original characteristics. In spite of this, the ectodermal differentiation of IFN-β-exposed K7-iPSCs was disrupted in a way similar to the process observed in IFNB-iPSCs. The results are in line with data by Aikawa and co-authors who used an in vitro blastocyst model and found that exposure to IFN-β reduced neither embryonic cell proliferation nor cell survival [72]. This suggests that IFN-β-dependent effects on iPSC ectodermal differentiation observed in our study are due to an alteration of the cell differentiation process rather than to an impairment of the initial pluripotent properties of iPSCs.

To the best of our knowledge, there is only one study that explored the effects of the IFN-I system on iPSC three-lineage differentiation. Eggenberger and co-authors [77] analyzed how the temporal induction of *IRF7* (a member of the interferon regulatory transcription factor family involved in the activation of IFN-I transcription) affected the iPSC transcriptional profile. They found the IRF7-dependent dysregulation of the expression of many genes in resting and differentiated iPSCs, primarily, the genes associated with endoderm and ectoderm development. Negative effects of IFN-β on ectoderm differentiation observed in our study are well in line with these results. However, we did not observe a stable effect of *IFNB* overexpression on endoderm- and mesoderm-associated genes, which contrasts with the results by Eggenberger and co-authors. The discrepancies may be due to several reasons. First, the studies differ by the methodology of IFN-β system induction (specifically, a constitutive overexpression of *IFNB* in our study and a transient induction of *IRF7* in the study by Eggenberger and co-authors). Second, we examined how *IFNB* overexpression affects the expression of only four endoderm genes; Eggenberger and co-authors analyzed the expression of multiple genes using the Taq-Man™ hPSC Scorecard assay. Third, in the two studies, iPSC differentiation was driven using different approaches, specifically, by culturing iPSCs in 3D low-adhesive serum-free conditions for 20 days in our study and by culturing cells in a monolayer in the presence of fetal calf serum (FCS) for 5 days in the study by Eggenberger and co-authors. Since, in both studies, the analysis was performed only at one point in time, the discrepancies may be not fundamental, and simply mirror the inter-study differences in the dynamics of gene expression. Whether or not the effect of IFN-β on endoderm and mesoderm lineages is confirmed in future studies, IFN-β-dependent impairment of iPSC differentiation into an ectoderm lineage seems to be very likely.

Of the five ectoderm-associated genes whose expression was analyzed in our study, only three genes, *PAX6*, *LHX2*, and *MAP2*, were reliably upregulated following iPSC differentiation. Of them, two genes, *PAX6* and *LHX2*, were reproducibly inhibited in IFNB-EBs and in K7-EBs differentiated in the presence of IFN-β. The arising questions are as follows: Why were not all the tested ectoderm-associated markers upregulated? And how reasonable is our conclusion on the inhibitory effect of IFN-β on neuroectoderm differentiation? To answer the questions, we need to briefly summarize data on each of the analyzed genes.

*PAX6* and *LHX2* are key transcriptional factors driving and marking neuroectoderm differentiation. *LHX2* is switched on before *PAX6*. *LHX2* determines neural induction by promoting the expression of *PAX6* and by inhibiting non-neural differentiation through the attenuation of BMP and WNT signaling pathways [64]. *PAX6* acts by repressing pluripotency genes and by activating neuroectoderm genes. It supports neural stem cell survival and self-renewal and induces the formation of progenitor cell populations and neural stem cell neurogenesis. The effects of *PAX6* are dosage-dependent: higher *PAX6* levels drive neural stem cell neurogenesis at the expense of self-renewal [79]. In an in vitro human ESC differentiation model, knockdown of *PAX6* blocked neuroectoderm formation [79]. Thus, it is likely that the IFN-β-mediated diminished expression of *LHX2* and *PAX6* observed in our study is biologically significant and, indeed, marks a disruption of neuroectoderm differentiation. This conclusion is in line with the well-documented neurotoxicity of type I IFNs [80,81,82,83], including recent data showing that maternal immune activation and an upregulation of IFN-β lead to behavioral abnormalities of the offspring [84]. The results also agree with recent data showing a disruption of iPSC neuronal differentiation in the presence of another interferon—IFN-γ [85].

OTX2 plays a pivotal role in brain morphogenesis, craniofacial development, and eye development, and it has been widely used as a marker of ectoderm differentiation, including the in vitro differentiation of pluripotent cells [86,87,88,89]. However, recent studies have demonstrated that undifferentiated iPSCs, in particular, those derived from peripheral mononuclear cells, already express OTX2 protein at a relatively high level [64,88,90]. These data corroborate well with our results. It was suggested that OTX2 cannot be used as a marker of neuroectoderm differentiation from iPSCs [90], and this notion may explain the fact that we did not detect the IFN-β-dependent downregulation of *OTX2* expression in our model. Another possible explanation of our data is that we performed a gene expression analysis at a time point before *OTX2* upregulation took place. Indeed, it was demonstrated that following human ESC differentiation, *OTX2* is upregulated later than *PAX6* [89].

SOX1 enhances neuroectodermal commitment and maintenance [91,92,93]. Like *OTX2*, *SOX1* is induced later than *LHX2* and *PAX6* [64], and this may explain the lack of *SOX1* induction and IFN-β-dependent inhibition in our study.

Our study has several limitations that need to be acknowledged. The main limitations are as follows: (i) for each of the three germ layers, we analyzed only a restricted number of marking genes; (ii) we performed a gene expression analysis at only one point in time. It should also be noted that a bulk analysis of the whole population of differentiating cells did not exclude the possibility that some cell types within the sample may have behaved differentially. However, the main focus of the study was to develop an *IFNB*-overexpressing model suitable for the analysis of IFN-β effects on different cell populations and differentiation processes. Dynamic transcriptomic and proteomic analyses, including the analysis at single-cell levels, are planned.

In conclusion, we have generated and characterized several lines of human iPSCs with a constitutive overexpression of *IFNB* and have documented their hampered differentiation into the ectodermal germ layer. To the best of our knowledge, this is the second published documentation of the disruption of stem cell ectodermal differentiation by the IFN-I system (after study [77]). The iPSC lines generated in our study represent a valuable model for exploring the effects of the active IFN-I system on early human embryoid development, and they provide a valuable cell source to generate various types of cells overexpressing IFN-β for future experimental and clinically oriented studies.

## 4. Materials and Methods

The suppliers and catalog numbers of all materials are presented in Table S1.

### 4.1. The Maintenance of iPSC Lines

iPSC line K7-4Lf (K7-iPSCs) previously generated from blood mononuclear cells of a healthy woman was used in the study [46]. The cells were tested for mycoplasma contamination using a PCR method (Mycoreport, Evrogen, Moscow, Russia; thermocycling conditions: preliminary denaturation at 95 °C for 10 min; 40 cycles of denaturation for 15 s; annealing at 62 °C for 15 s; elongation at 72 °C for 15 s). If not indicated otherwise, the cells were maintained on mouse embryo fibroblasts (MEFs) in an iPSC medium consisting of a DMEM/F12 medium (Gibco, Waltham, MA, USA) supplemented with 15% KnockOut Serum Replacement, 1% non-essential amino acids, 1% penicillin/streptomycin, 1% GlutaMAX Supplement (all from Thermo Fisher Scientific, Waltham, MA, USA), 0.055 mM β-mercaptoethanol (Sigma-Aldrich, St. Louis, MO, USA), and fibroblast growth factor-2 (FGF-2; 10 ng/mL; SCI Store). The medium was replaced daily. For passages, the wells were washed with phosphate-buffered saline (PBS), cell colonies were disrupted using a TrypLE Express Enzyme (Thermo Fisher Scientific, Waltham, MA, USA), and the cells were transferred into new wells at a 1:5–1:10 split ratio. For the first 24 h after the passaging, thiazovivin, an inhibitor of the Rho-associated coiled-coil-containing protein kinase (ROCK), was added to the cultures (final concentration, 10 µM; Sigma-Aldrich). For some purposes (i.e., for karyotyping and immunofluorescence analysis), iPSCs were depleted of MEFs. For that, the cells were cultured for 2–3 passages in feeder-free conditions in an mTeSR™1 medium (STEMCELL Technologies, Vancouver, BC, Canada) on Matrigel (Corning, NY, USA).

### 4.2. The Generation of Plasmid Expressing Human IFNB

The nucleotide sequence encoding interferon beta 1 (IFNB1, NM_002176.4) was synthesized by PCR using Q5 High-Fidelity DNA Polymerase (New England Biolabs, Ipswich, MA, USA), human genomic DNA as a template, and primer pairs IFNB1-F-CciNI and IFNB1-R-PmeI containing sites of restriction endonucleases CciNI (SibEnzyme, Novosibirsk, Russia) and PmeI (New England Biolabs), respectively. To generate the pAAVS1-hPGK-IFNB1 plasmid, the IFNB1 PCR product was cloned into the AAVS1_Puro_PGK1_3xFLAG_Twin_Strep vector (Addgene, #68375, Watertown, MA, USA) using the CciNI and PmeI restriction sites. The correct assembly of the plasmids was confirmed by a restriction analysis and Sanger sequencing of plasmid DNA (Institute of Chemical Biology and Fundamental Medicine SB RAS, SB RAS Genomics Core Facility, Novosibirsk, Russia).

### 4.3. The Generation of iPSC Lines Overexpressing IFNB

To generate iPSCs overexpressing the IFNB gene, the K7-iPSC line was used as a parental one. The cells (mycoplasma-tested, passage 10) were expanded to reach a confluence level of 70% and passaged on MEF-coated Petri dishes (Corning, Corning, NY, USA). On the next day, the cells were dissociated using the TrypLE Express Enzyme (Thermo Fisher Scientific), and counted, and 10^6^ cells were transfected using the Neon™ Transfection System 100 μL Kit (Invitrogen, Waltham, MA, USA) according to the manufacturer’s instructions (Neon™ NxT Electroporation System, Invitrogen™, Thermo Fisher Scientific, Waltham, MA, USA; one pulse, 30 ms, 1100 V). The plasmids used for the transfection were as follows: pAAVS1-hPGK-IFNB1 (expressing the human IFNB ORF) and pX458-AAVS1 (Addgene, expressing SpCas9 nuclease, EGFP, and sgRNA targeting the human AAVS1 locus [94]). After the transfection, the cells were seeded on MEF-coated 10 cm^2^ Petri dishes (Corning) in the iPSC medium containing thiazovivin but without the addition of penicillin/streptomycin. For the selection of transfected iPSC clones, puromycin dihydrochloride (300 ng/mL; AppliChem, Omaha, NE, USA) was added to the iPSC medium. After 5 days of culture, the medium was replaced with a standard one containing penicillin/streptomycin. The clones that survived puromycin selection were manually harvested, individually transferred into the wells of 48-well plates (Corning), and expanded. Genomic DNA was extracted from growing iPSC clones using a QuickExtract™DNA Extraction Solution (Lucigen, Middleton, WI, USA) and analyzed for the presence of the following: (i) wild-type AAVS1 loci not containing a target insert; (ii) target inserts of the donor plasmid containing the ORF of the human IFNB gene; and (iii) off-target inserts of the donor plasmid. The analysis included PCR followed by polyacrylamide gel electrophoresis. For PCR, the following primers were used: (i) AAVS1_WT-F and AAVS1_WT-R (wild-type locus detection); (ii) HA_L_OUT and Puro_in-R (target insert detection); (iii) pRS-marker and Puro_in-R (unincorporated donor plasmid DNA detection; for the sequences of the primers, see Table S2). iPSC clones containing target inserts and lacking off-target inserts were expanded and referred to as IFNB-iPSCs.

### 4.4. Karyotyping Analysis

Karyotyping of IFNB-iPSC lines was performed at passage 11 as previously described [95], with some modifications. In brief, the cells were treated with Colcemid (0.05 μg/mL; Biolot, Saint Petersburg, Russia), washed with 10 mM PBS, dissociated with TrypLE (3 min, room temperature), and incubated in a hypotonic solution of KCl (0.28%, 37 °C, 20 min). For prefixation, 2–3 drops of Carnoy’s fixative (3:1, methanol/acetic acid) were added to the KCl solution. The cells were carefully resuspended, pelleted (270 g, 7 min), and incubated in ice-cold Carnoy’s fixative (15 min on ice). The cycle was repeated twice. Before G-banding, fresh ice-cold Carnoy’s fixative was added to the cells and the cell suspension was dripped onto cooled slides and dried. The preparations were stained in 5% Giemsa solution for 10–20 min. The chromosome G-banding analysis was performed using the International System for Human Cytogenetic Nomenclature. Twenty metaphase plates were analyzed.

### 4.5. Spontaneous Differentiation of iPSC Lines

The pluripotency of IFN-iPSCs was evaluated by generating embryoid bodies (EBs) and evaluating their capacity to spontaneously form endoderm, mesoderm, and ectoderm germ layers. iPSCs were treated with collagenase IV (1 mg/mL, 37 °C, 5–10 min, Gibco); cell aggregates were dislodged by scraping, transferred to ultra-low-adhesive 6-well plates (Corning) or tissue-culture plates coated with 1% agarose (Sigma-Aldrich), and cultured in the iPSC medium without the addition of FGF2. The medium was refreshed on days 2 and 5 and then every 5 days until day 15 (immunofluorescence analysis) or day 21 (RT-PCR analysis).

To evaluate the effect of exogenous IFN-β on iPSC spontaneous differentiation, recombinant human IFN-β was added to some of the K7-iPSC cultures (500 ng/mL; APA222Hu01, Cloud-Clone Corp., Houston, TX, USA). EBs were maintained and collected as described above.

### 4.6. Alkaline Phosphatase Staining and Immunofluorescence Analysis

The detection of endogenous alkaline phosphatase in iPSCs was performed with the Leukocyte Alkaline Phosphatase kit (Sigma-Aldrich) according to the manufacturer protocol. Briefly, the cells were washed with PBS, fixed in a citrate–acetone–formaldehyde fixative, washed, stained with an alkaline-dye mixture (15 min in the dark), and rinsed with deionized water.

The immunofluorescence analysis included the evaluation of pluripotency markers in iPSCs and endoderm-, mesoderm-, and ectoderm-associated markers in spontaneously differentiated EBs. EBs were generated as described above. On day 15, EBs were transferred from low-adhesive conditions to Matrigel-coated coverglasses to allow EBs to spread out; 6 days later, the EBs were collected. For the immunofluorescence analysis, the prepared iPSCs and EBs were fixed in 4% paraformaldehyde (room temperature; 10 min and 20 min for iPSCs and EBs, respectively; Sigma-Aldrich) and incubated with a blocking buffer consisting of PBS supplemented with 10% sheep serum (Sigma-Aldrich), 1% bovine serum albumin (Sigma-Aldrich), 1% Triton-X100, and 0.05% NaN3 (room temperature; 1 h for iPSCs and 2 h for EBs). The cells were then incubated with primary antibodies (4 °C; overnight for iPSCs and 2 days for EBs), washed intensively (room temperature; orbital shaker; 20 min, 3 times), incubated with fluorescent dye-labeled secondary antibodies (room temperature; 2 h), and washed. For the analysis of pluripotency marker expression, the following pairs of antibodies were used: (i) rabbit-anti-OCT4 (1:200, Cambridge) and goat-anti-rabbit Alexa Fluor 568 (1:1000, Invitrogen) and (ii) rabbit-anti-SOX2 (1:200, Abclonal, Woburn, MA, USA) and goat-anti-rabbit Alexa Fluor 488 (1:1000, Invitrogen). For EB staining, the following antibodies specific to endoderm, mesoderm, and ectoderm markers were used: rabbit-anti-human AFP (1:200; China) and goat-anti-rabbit Alexa Fluor 546 (1:200, Thermo Fischer Scientific); rabbit-anti-human ACTA2 (1:100, Abclonal, Woburn, MA, USA) and goat-anti-rabbit Alexa Fluor 546 (1:200, Thermo Fischer Scientific); and mouse-anti-human TUBB3 (1:100, Abclonal) and donkey-anti-mouse Alexa Fluor 488 (1:200, Thermo Fischer Scientific). Cells were stained with DAPI (4′,6-diamidino-2-phenylindol; BioLegend, San Diego, CA, USA) before they were mounted and immersed into a Dako fluorescent mounting medium (Agilent, Santa Clara, CA, USA).

### 4.7. Real-Time PCR

RNA was isolated by using the RNeasy Mini kit (pluripotency marker expression analysis; QIAGEN, Venlo, The Netherlands) or the total RNA extraction kit (ISG expression analysis; ELK Biotechnology, Amersham, Slough, Buckinghamshire, UK) or by manually using the ExtractRNA reagent (three germ layer gene expression analyses; Evrogen, Moscow, Russia). RNA from experimental (e.g., IFNB-iPSCs, IFNB-EBs) and control (K7-iPSCs, K7-EBs) cells was isolated simultaneously using the same RNA isolation procedure. RNA concentrations were determined on a NanoDrop one spectrophotometer (Thermo Fischer Scientific); all RNAs were equalized in concentration, aliquoted, and stored at −80 °C. Reverse transcription was performed using the M-MuLV–RH first strand cDNA synthesis kit (Biolabmix, Novosibirsk, Russia) according to the manufacturer’s instructions and the following thermocycling conditions: 25 °C, 10 min; 42 °C, 60 min; 70 °C, 10 min; and holding at 4 °C. Real-time PCR was performed using the HS Taq DNA polymerase kit (Evrogen) and Light-Cycler 480 Real-Time PCR system (Roche, Basel, Switzerland) using the following PCR procedure: initial denaturation, 95 °C for 60 s; and 40 cycles of denaturation–annealing, 95 °C for 15 s and 60 °C for 60 s, respectively. The following conditions were applied for a melt curve analysis, 95 °C, 15 s; 60 °C, 60 s; and 95 °C, 15 s. The results were analyzed using the LightCycler 96 program. Ct-values were normalized to the human *RPL27* and *GAPDH* genes using the ΔΔCq-method [96] (the housekeeping genes were chosen based on the results of preliminary experiments analyzing the stability of gene expression following iPSC-to-EB differentiation). Primers and fluorescently labeled probes were purchased from DNA-Synthesis (Table S2).

### 4.8. Western Blotting

The expressions of IFN-β, PAX6, and OCT4 at the protein level were evaluated using Western blotting and β-actin (IFN-β) or HSP90 (PAX6, OCT4) as normalization proteins. The procedure was performed as described earlier [39]. Briefly, IFNB-iPSCs and parental K7-iPSCs (2 × 10^6^ cells in each case, respectively) were washed in PBS, pelleted, and stored at −80 °C. The pellets were lysed with an RIPA Lysis and Extraction Buffer (Thermo Fischer Scientific) in the presence of a cOmplete™ protease inhibitor cocktail (Roche) and centrifuged (12,000× *g*, 40C, 20 min). After the determination of protein concentration (Pierce™ BCA Protein Assay Kit, Thermo Fischer Scientific), 60 µg of proteins was electrophoresed in a 12% SDS/polyacrylamide gel and transferred onto a 0.45 µm nitrocellulose NitroPure™ membrane (GVS Life Sciences, Bologna, Italy). The membrane was blocked with a 5% non-fat dry milk (Cell Signaling Technology, Danvers, MA, USA) in a TNT buffer (10 mM of Tris-HCl, pH 7.5, 150 mM of NaCl, 0.1% Tween-20) and incubated with the following primary antibodies (4 °C, overnight, 1% milk): rabbit anti-human-IFN-β polyclonal antibodies (1:500, FNab10475, FineTest Biotech, Wuhan, Hubei, China), rabbit anti-human-PAX6 polyclonal antibodies (1:500, PAH446Ra01, Cloud-Clone Corp.), mouse anti-human-OCT-4 monoclonal antibody (1:1000, ab184665, Abcam, Cambridge, UK), mouse anti-human-actin-β antibodies (1:10,000, Abcam); and rabbit anti-human-HSP90 polyclonal antibodies (1:10,000, Sigma-Aldrich). Thereafter, the membrane was washed and incubated with horseradish peroxidase-conjugated polyclonal goat anti-rabbit IgG (1:10,000, Jackson ImmunoResearch, West Grove, PA, USA) or polyclonal goat anti-mouse IgG (1:2000, Biolegend, San Diego, California, USA.; 2 h, room temperature). Chemiluminescence was detected using an ECL Western Blotting Detection Kit (Amersham, Slough, Buckinghamshire, UK).

### 4.9. ELISA

Supernatants were obtained from IFNB-iPSC and parental K7-iPSC cultures, aliquoted, and stored at −80 °C until the day of the analysis. Culture supernatants were analyzed using a Human Interferon-β ELISA Kit (CSB-E09889, Cusabio, Houston, TX, USA) according to the manufacturer’s instructions using microplate spectrophotometer Thermo Scientific™ Multiscan™ GO and ScanIt™ Software v7.1 (Thermo Fisher Scientific, Waltham, MA, USA).

### 4.10. Functional Analysis

To test the functional activity of IFN-β secreted by IFNB-iPSCs, we analyzed the ability of IFNB-iPSC-derived supernatants to induce the expression of ISGs in human macrophage-like cells. The supernatants were collected from the feeder-free cultures of IFNB-iPSC lines LA8 and LC8 and from the parental K7-iPSCs. The supernatants were centrifuged to eliminate possible cell contamination and stored at −70 °C.

THP-1 cells (a human monocyte-like cell line, a kind gift by Dr. V. Tatarskiy) were cultured in the wells of 12-well plates in an RPMI-1640 medium (Biowest, Bradenton, FL, USA) supplemented with 10% FCS (HyClone), 1% penicillin/streptomycin, 1% GlutaMAX Supplement, 1% non-essential amino acids (all from Thermo Fisher Scientific) and 0.055 mM β-mercaptoethanol (Sigma-Aldrich). The differentiation of THP-1 monocytes into macrophage-like cells was stimulated by adding phorbol 12-myristate-13-acetate (PMA; 100 ng/mL, Sigma-Aldrich). Four days after PMA stimulation, half of the medium (0.5 mL per well) was carefully aspirated from the cultures in such a way as not to disturb the cultured cells and was substituted with the supernatants obtained from IFNB-iPSCs, the supernatants obtained from K7-iPSCs, or a fresh medium. After 24h culture, THP-1 cells were lysed using the ExtractRNA reagent (Evrogen), and RNA was isolated and used to evaluate the expression of ISGs in THP-1 macrophages using RT-qPCR.

### 4.11. Directed Ectodermal Differentiation of iPSCs

To induce directed ectoderm differentiation, on day 0, iPSCs grown on Matrigel were dissociated, counted, and passaged to the Matrigel-coated wells of a 12-well plate in the mTeSR™1 medium supplemented with 1% penicillin/streptomycin and thiazovivin. On the next day, the mTeSR™1 medium was substituted with the STEMdiff™ Trilineage Ectoderm Medium (StemCell Technologies). The starting cell quantities were 4 × 10^5^ cells/well. The medium was refreshed every day until day 7, when the cells were lysed for RNA isolation (ExtractRNA reagent, Evrogen) and the RT-PCR analysis.

### 4.12. Evaluation of Cell Viability Using Flow Cytometry

To assess the viability of IFNB-iPSCs and K7-iPSCs, iPSC cultures were treated with the TrypLE Express Enzyme (Thermo Fisher Scientific), washed, and stained using the LIVE/DEAD™ Fixable Far Red Dead Cell Stain Kit (L34974, Invitrogen™). The cells were analyzed on a CytoFLEX-S cytometer (Beckman Coulter, Brea, CA, USA) using CytEXPERT software version 2.4.0.28 (Beckman Coulter). The results were analyzed using FlowJo^TM^ software v10.8.1 (TreeStar BD Bioscience, Franklin Lakes, NJ, USA).

### 4.13. Statistical Analysis

Differences between the groups were analyzed using the nonparametric Kruskal–Wallis test; correction for multiple comparisons was performed by determining the false discovery rate (q-value) using the two-stage step-up method of Benjamini, Krieger, and Yekutieli (GraphPad Prism 8.0.1 Software Inc., https://www.graphpad.com/, San Diego, CA, USA). For some data, the Mann–Whitney test was also performed. A q-value < 0.05 was considered to be statistically significant. The graphs show boxes and whiskers with minimum and maximum values. For the results with n ≤ 3, individual data are also shown. For each experiment, the number of experimental repeats is indicated in the figure legends.

## 5. Conclusions

In this study, we describe the generation and the characterization of human iPSC lines overexpressing *IFNB*. The main outcomes of the studies are as follows: (i) it is feasible to generate IFNB-overexpressing iPSCs; (ii) IFNB-overexpressing iPSCs display the main characteristics of pluripotency; (iii) IFN-β hampers iPSC neuroectoderm differentiation. IFNB-overexpressing iPSC lines generated in the study create an in vitro model for an in-depth analysis of the effects of the IFN-I system on early human embryonic development; they also provide a valuable cell source to generate various types of differentiated cells overexpressing IFN-β for future experimental and clinically oriented studies.

## Data Availability

The raw data supporting the conclusions of this article will be made available by the authors on request.

## Supplementary Materials

The following supporting information can be downloaded at: https://www.mdpi.com/article/10.3390/ijms252212456/s1.

## Abbreviations

ACTA2: actin alpha 2; AFP, alpha-fetoprotein; DAPI, 4′,6-diamidino-2-phenylindol; EBs, embryoid bodies; ESCs, embryonic stem cells; FCS, fetal calf serum; FGF-2, fibroblast growth factor-2; GATA4, GATA-binding protein 4; HAND1, heart and neural crest-derived transcript 1; HHEX, haematopoietically expressed homeobox; IFN-I, type I interferon; IFNAR, interferon I receptor; IFNB-EBs, embryoid bodies derived from IFNB-iPSCs; IFNB-iPSCs, iPSC cell lines with a constitutive overexpression of the IFNB gene; IRFs, interferon regulatory factors; ISGs, interferon-stimulated genes; K7-EBs, embryoid bodies derived from K7-iPSCs; K7-iPSCs, K7-4Lf iPSC line (alternative name: ICGi022-A); LHX2, LIM homeobox 2; MAP2, microtubule-associated protein 2; MEFs, mouse embryo fibroblasts; MX1, myxovirus resistance 1; OAS, 2′-5′-oligoadenylate synthetase 1; OCT4, octamer-binding transcription factor 4; OTX2, orthodenticle homeobox 2; PAX6, paired box 6; PBS, phosphate-buffered saline; PMA, phorbol 12-myristate-13-acetate; SNAI2, snail family transcriptional repressor 2; SOX, SRY-Box transcription factor; TBXT, T-box transcription factor T; TUBBIII, tubulin beta-3 Class III.

## References

[1] Shi Y., Inoue H., Wu J.C., Yamanaka S. (2017). Induced Pluripotent Stem Cell Technology: A Decade of Progress. Nat. Rev. Drug Discov..

[2] Liu G., David B.T., Trawczynski M., Fessler R.G. (2020). Advances in Pluripotent Stem Cells: History, Mechanisms, Technologies, and Applications. Stem Cell Rev. Rep..

[3] Schoggins J.W. (2019). Interferon-Stimulated Genes: What Do They All Do?. Annu. Rev. Virol..

[4] Lazear H.M., Schoggins J.W., Diamond M.S. (2019). Shared and Distinct Functions of Type I and Type III Interferons. Immunity.

[5] Sadler A.J., Williams B.R. (2008). Interferon-Inducible Antiviral Effectors. Nat. Rev. Immunol..

[6] McNab F., Mayer-Barber K., Sher A., Wack A., O’garra A. (2015). Type I Interferons in Infectious Disease. Nat. Rev. Immunol..

[7] Crouse J., Kalinke U., Oxenius A. (2015). Regulation of Antiviral T Cell Responses by Type I Interferons. Nat. Rev. Immunol..

[8] Kumar A., Khani A.T., Swaminathan S. (2021). Type I Interferons: One Stone to Concurrently Kill Two Birds, Viral Infections and Cancers. Curr. Res. Virol. Sci..

[9] Decker T., Müller M., Stockinger S. (2005). The Yin and Yang of Type I Interferon Activity in Bacterial Infection. Nat. Rev. Immunol..

[10] Boxx G.M., Cheng G. (2016). The Roles of Type I Interferon in Bacterial Infection. Cell Host Microbe.

[11] Kovarik P., Castiglia V., Ivin M., Ebner F. (2016). Type I Interferons in Bacterial Infections: A Balancing. Act. Front. Immunol..

[12] Peignier A., Parker D. (2021). Impact of Type I Interferons on Susceptibility to Bacterial Pathogens. Trends Microbiol..

[13] Auerbuch V., Brockstedt D.G., Meyer-Morse N., O’Riordan M., Portnoy D.A. (2004). Mice Lacking the Type I Interferon Receptor Are Resistant to Listeria Monocytogenes. J. Exp. Med..

[14] Carrero J.A., Calderon B., Unanue E.R. (2004). Type I Interferon Sensitizes Lymphocytes to Apoptosis and Reduces Resistance to Listeria Infection. J. Exp. Med..

[15] Qiu H., Fan Y., Joyee A.G., Wang S., Han X., Bai H., Jiao L., Van Rooijen N., Yang X. (2008). Type I IFNs Enhance Susceptibility to Chlamydia Muridarum Lung Infection by Enhancing Apoptosis of Local Macrophages. J. Immunol..

[16] Shahangian A., Chow E.K., Tian X., Kang J.R., Ghaffari A., Liu S.Y., Belperio J.A., Cheng G., Deng J.C. (2009). Type I IFNs Mediate Development of Postinfluenza Bacterial Pneumonia in Mice. J. Clin. Investig..

[17] Dorhoi A., Yeremeev V., Nouailles G., Weiner J., Jörg S., Heinemann E., Oberbeck-Müller D., Knaul J.K., Vogelzang A., Reece S.T. (2014). Type I IFN Signaling Triggers Immunopathology in Tuberculosis-susceptible Mice by Modulating Lung Phagocyte Dynamics. Eur. J. Immunol..

[18] Zhu Q., Man S.M., Karki R., Malireddi R.S., Kanneganti T.-D. (2018). Detrimental Type I Interferon Signaling Dominates Protective AIM2 Inflammasome Responses during Francisella Novicida Infection. Cell Rep..

[19] Gresser I., Bourali C., Lévy J.P., Fontaine-Brouty-Boyé D., Thomas M.T. (1969). Increased survival in mice inoculated with tumor cells and treated with interferon preparations. Proc. Natl. Acad. Sci. USA.

[20] Schiavoni G., Mattei F., Gabriele L. (2013). Type I Interferons as Stimulators of DC-Mediated Cross-Priming: Impact on Anti-Tumor Response. Front. Immunol..

[21] Escobar G., Moi D., Ranghetti A., Ozkal-Baydin P., Squadrito M.L., Kajaste-Rudnitski A., Bondanza A., Gentner B., De Palma M., Mazzieri R. (2014). Genetic Engineering of Hematopoiesis for Targeted IFN-α Delivery Inhibits Breast Cancer Progression. Sci. Transl. Med..

[22] Demaria O., De Gassart A., Coso S., Gestermann N., Di Domizio J., Flatz L., Gaide O., Michielin O., Hwu P., Petrova T.V. (2015). STING Activation of Tumor Endothelial Cells Initiates Spontaneous and Therapeutic Antitumor Immunity. Proc. Natl. Acad. Sci. USA.

[23] Hirata A., Hashimoto H., Shibasaki C., Narumi K., Aoki K. (2019). Intratumoral IFN-α Gene Delivery Reduces Tumor-Infiltrating Regulatory T Cells through the Downregulation of Tumor CCL17 Expression. Cancer Gene Ther..

[24] Zhou L., Zhang Y., Wang Y., Zhang M., Sun W., Dai T., Wang A., Wu X., Zhang S., Wang S. (2020). A Dual Role of Type I Interferons in Antitumor Immunity. Adv. Biosyst..

[25] Shi W., Yao X., Fu Y., Wang Y. (2022). Interferon-α and Its Effects on Cancer Cell Apoptosis. Oncol. Lett..

[26] Razaghi A., Durand-Dubief M., Brusselaers N., Björnstedt M. (2023). Combining PD-1/PD-L1 Blockade with Type I Interferon in Cancer Therapy. Front. Immunol..

[27] Yu R., Zhu B., Chen D. (2022). Type I Interferon-Mediated Tumor Immunity and Its Role in Immunotherapy. Cell. Mol. Life Sci..

[28] Huang L., Li L., Lemos H., Chandler P.R., Pacholczyk G., Baban B., Barber G.N., Hayakawa Y., McGaha T.L., Ravishankar B. (2013). Cutting Edge: DNA Sensing via the STING Adaptor in Myeloid Dendritic Cells Induces Potent Tolerogenic Responses. J. Immunol..

[29] Zitvogel L., Galluzzi L., Kepp O., Smyth M.J., Kroemer G. (2015). Type I Interferons in Anticancer Immunity. Nat. Rev. Immunol..

[30] Koba C., Haruta M., Matsunaga Y., Matsumura K., Haga E., Sasaki Y., Ikeda T., Takamatsu K., Nishimura Y., Senju S. (2013). Therapeutic Effect of Human iPS-Cell–Derived Myeloid Cells Expressing IFN-β against Peritoneally Disseminated Cancer in Xenograft Models. PLoS ONE.

[31] Senju S., Koba C., Haruta M., Matsunaga Y., Matsumura K., Haga E., Sasaki Y., Ikeda T., Takamatsu K., Nishimura Y. (2014). Application of iPS Cell-Derived Macrophages to Cancer Therapy. OncoImmunology.

[32] Miyashita A., Fukushima S., Nakahara S., Kubo Y., Tokuzumi A., Yamashita J., Aoi J., Haruta M., Senju S., Nishimura Y. (2016). Immunotherapy against Metastatic Melanoma with Human iPS Cell–Derived Myeloid Cell Lines Producing Type I Interferons. Cancer Immunol. Res..

[33] Tsuchiya N., Zhang R., Iwama T., Ueda N., Liu T., Tatsumi M., Sasaki Y., Shimoda R., Osako Y., Sawada Y. (2019). Type I Interferon Delivery by iPSC-Derived Myeloid Cells Elicits Antitumor Immunity via XCR1+ Dendritic Cells. Cell Rep..

[34] Swiecki M., Colonna M. (2011). Type I Interferons: Diversity of Sources, Production Pathways and Effects on Immune Responses. Curr. Opin. Virol..

[35] Ali S., Mann-Nüttel R., Schulze A., Richter L., Alferink J., Scheu S. (2019). Sources of Type I Interferons in Infectious Immunity: Plasmacytoid Dendritic Cells Not Always in the Driver’s Seat. Front. Immunol..

[36] Park M.D., Silvin A., Ginhoux F., Merad M. (2022). Macrophages in Health and Disease. Cell.

[37] Weiss G., Schaible U.E. (2015). Macrophage Defense Mechanisms against Intracellular Bacteria. Immunol. Rev..

[38] Banete A., Barilo J., Whittaker R., Basta S. (2022). The Activated Macrophage–A Tough Fortress for Virus Invasion: How Viruses Strike Back. Front. Microbiol..

[39] Mantovani A., Allavena P., Marchesi F., Garlanda C. (2022). Macrophages as Tools and Targets in Cancer Therapy. Nat. Rev. Drug Discov..

[40] Klepikova A., Nenasheva T., Sheveleva O., Protasova E., Antonov D., Gainullina A., Chikina E., Sakovnich O., Gerasimova T., Nikitina I. (2022). iPSC-Derived Macrophages: The Differentiation Protocol Affects Cell Immune Characteristics and Differentiation Trajectories. Int. J. Mol. Sci..

[41] Sheveleva O., Protasova E., Nenasheva T., Butorina N., Melnikova V., Gerasimova T., Sakovnich O., Kurinov A., Grigor’eva E.V., Medvedev S.P. (2023). A Model of iPSC-Derived Macrophages with TNFAIP3 Overexpression Reveals the Peculiarities of TNFAIP3 Protein Expression and Function in Human Macrophages. Int. J. Mol. Sci..

[42] Lee C.Z., Kozaki T., Ginhoux F. (2018). Studying Tissue Macrophages in Vitro: Are iPSC-Derived Cells the Answer?. Nat. Rev. Immunol..

[43] Lyadova I., Gerasimova T., Nenasheva T. (2021). Macrophages Derived from Human Induced Pluripotent Stem Cells: The Diversity of Protocols, Future Prospects, and Outstanding Questions. Front. Cell Dev. Biol..

[44] Gutbier S., Wanke F., Dahm N., Rümmelin A., Zimmermann S., Christensen K., Köchl F., Rautanen A., Hatje K., Geering B. (2020). Large-Scale Production of Human iPSC-Derived Macrophages for Drug Screening. Int. J. Mol. Sci..

[45] Ackermann M., Kempf H., Hetzel M., Hesse C., Hashtchin A.R., Brinkert K., Schott J.W., Haake K., Kühnel M.P., Glage S. (2018). Bioreactor-Based Mass Production of Human iPSC-Derived Macrophages Enables Immunotherapies against Bacterial Airway Infections. Nat. Commun..

[46] Malakhova A.A., Grigor’eva E.V., Pavlova S.V., Malankhanova T.B., Valetdinova K.R., Vyatkin Y.V., Khabarova E.A., Rzaev J.A., Zakian S.M., Medvedev S.P. (2020). Generation of Induced Pluripotent Stem Cell Lines ICGi021-A and ICGi022-A from Peripheral Blood Mononuclear Cells of Two Healthy Individuals from Siberian Population. Stem Cell Res..

[47] Ustyantseva E., Pavlova S.V., Malakhova A.A., Ustyantsev K., Zakian S.M., Medvedev S.P. (2022). Oxidative Stress Monitoring in iPSC-Derived Motor Neurons Using Genetically Encoded Biosensors of H_2_O_2_. Sci. Rep..

[48] Wei L., Sandbulte M.R., Thomas P.G., Webby R.J., Homayouni R., Pfeffer L.M. (2006). NFκB Negatively Regulates Interferon-Induced Gene Expression and Anti-Influenza Activity. J. Biol. Chem..

[49] Pervolaraki K., Stanifer M.L., Münchau S., Renn L.A., Albrecht D., Kurzhals S., Senís E., Grimm D., Schröder-Braunstein J., Rabin R.L. (2017). Type I and Type III Interferons Display Different Dependency on Mitogen-Activated Protein Kinases to Mount an Antiviral State in the Human Gut. Front. Immunol..

[50] Itskovitz-Eldor J., Schuldiner M., Karsenti D., Eden A., Yanuka O., Amit M., Soreq H., Benvenisty N. (2000). Differentiation of Human Embryonic Stem Cells into Embryoid Bodies Comprising the Three Embryonic Germ Layers. Mol. Med..

[51] Sheridan S.D., Surampudi V., Rao R.R. (2012). Analysis of Embryoid Bodies Derived from Human Induced Pluripotent Stem Cells as a Means to Assess Pluripotency. Stem Cells Int..

[52] De Los Angeles A., Ferrari F., Xi R., Fujiwara Y., Benvenisty N., Deng H., Hochedlinger K., Jaenisch R., Lee S., Leitch H.G. (2015). Hallmarks of Pluripotency. Nature.

[53] Stover A.E., Schwartz P.H., Schwartz P.H., Wesselschmidt R.L. (2011). The Generation of Embryoid Bodies from Feeder-Based or Feeder-Free Human Pluripotent Stem Cell Cultures. Human Pluripotent Stem Cells.

[54] Lamba D.A., McUsic A., Hirata R.K., Wang P.-R., Russell D., Reh T.A. (2010). Generation, Purification and Transplantation of Photoreceptors Derived from Human Induced Pluripotent Stem Cells. PLoS ONE.

[55] Shafa M., Yang F., Fellner T., Rao M.S., Baghbaderani B.A. (2018). Human-Induced Pluripotent Stem Cells Manufactured Using a Current Good Manufacturing Practice-Compliant Process Differentiate into Clinically Relevant Cells from Three Germ Layers. Front. Med..

[56] Mora C., Serzanti M., Giacomelli A., Beltramone S., Marchina E., Bertini V., Piovani G., Refsgaard L., Olesen M.S., Cortellini V. (2017). Generation of Induced Pluripotent Stem Cells (iPSC) from an Atrial Fibrillation Patient Carrying a PITX2 p. M200V Mutation. Stem Cell Res..

[57] Ustyantseva E.I., Medvedev S.P., Vetchinova A.S., Illarioshkin S.N., Leonov S.V., Zakian S.M. (2020). Generation of an Induced Pluripotent Stem Cell Line, ICGi014-A, by Reprogramming Peripheral Blood Mononuclear Cells from a Patient with Homozygous D90A Mutation in SOD1 Causing Amyotrophic Lateral Sclerosis. Stem Cell Res..

[58] Bono F., Mutti V., Piovani G., Minelli A., Mingardi J., Guglielmi A., Missale C., Gennarelli M., Fiorentini C., Barbon A. (2021). Establishment and Characterization of Induced Pluripotent Stem Cell (iPSCs) Line UNIBSi014-A from a Healthy Female Donor. Stem Cell Res..

[59] Ju Z.-H., Liang X., Ren Y.-Y., Shu L.-W., Yan Y.-H., Cui X. (2021). Neurons Derived from Human-Induced Pluripotent Stem Cells Express Mu and Kappa Opioid Receptors. Neural Regen. Res..

[60] Drozd A.M., Walczak M.P., Piaskowski S., Stoczynska-Fidelus E., Rieske P., Grzela D.P. (2015). Generation of Human iPSCs from Cells of Fibroblastic and Epithelial Origin by Means of the oriP/EBNA-1 Episomal Reprogramming System. Stem Cell Res. Ther..

[61] Lu V., Doan M.T., Roy I.J., Torres A., Teitell M.A. (2022). Protocol for Germ Lineage Differentiation of Primed Human Pluripotent Stem Cells Using Chemically Defined, Nutrient-Balanced Media. STAR Protoc..

[62] Feng Z., Yang X., Guan J., Song W., Liu Y. (2023). Establishment of an Induced Pluripotent Stem Cell Line SDQLCHi048-A from a Healthy Boy Donor. Stem Cell Res..

[63] Kim M.-H., Thanuthanakhun N., Kino-Oka M. (2024). Stable and Efficient Generation of Functional iPSC-Derived Neural Progenitor Cell Rosettes through Regulation of Collective Cell-Cell Behavior. Front. Bioeng. Biotechnol..

[64] Hou P.-S., Chuang C.-Y., Kao C.-F., Chou S.-J., Stone L., Ho H.-N., Chien C.-L., Kuo H.-C. (2013). LHX2 Regulates the Neural Differentiation of Human Embryonic Stem Cells via Transcriptional Modulation of PAX6 and CER1. Nucleic Acids Res..

[65] Yamanaka S. (2020). Pluripotent Stem Cell-Based Cell Therapy—Promise and Challenges. Cell Stem Cell.

[66] Jiang Z., Han Y., Cao X. (2014). Induced Pluripotent Stem Cell (iPSCs) and Their Application in Immunotherapy. Cell. Mol. Immunol..

[67] Chehelgerdi M., Behdarvand Dehkordi F., Chehelgerdi M., Kabiri H., Salehian-Dehkordi H., Abdolvand M., Salmanizadeh S., Rashidi M., Niazmand A., Ahmadi S. (2023). Exploring the Promising Potential of Induced Pluripotent Stem Cells in Cancer Research and Therapy. Mol. Cancer.

[68] Cerneckis J., Cai H., Shi Y. (2024). Induced Pluripotent Stem Cells (iPSCs): Molecular Mechanisms of Induction and Applications. Signal Transduct. Target. Ther..

[69] Ávila-González D., Gidi-Grenat M.Á., García-López G., Martínez-Juárez A., Molina-Hernández A., Portillo W., Díaz-Martínez N.E., Díaz N.F. (2023). Pluripotent Stem Cells as a Model for Human Embryogenesis. Cells.

[70] Weatherbee B.A., Gantner C.W., Iwamoto-Stohl L.K., Daza R.M., Hamazaki N., Shendure J., Zernicka-Goetz M. (2023). Pluripotent Stem Cell-Derived Model of the Post-Implantation Human Embryo. Nature.

[71] Chen L.L., Yang L., Carmichael G.G. (2010). Molecular basis for an attenuated cytoplasmic dsRNA response in human embryonic stem cells. Cell Cycle.

[72] Aikawa H., Tamai M., Mitamura K., Itmainati F., Barber G.N., Tagawa Y. (2014). Innate Immunity in an in Vitro Murine Blastocyst Model Using Embryonic and Trophoblast Stem Cells. J. Biosci. Bioeng..

[73] Wang R., Teng C., Spangler J., Wang J., Huang F., Guo Y.-L. (2014). Mouse Embryonic Stem Cells Have Underdeveloped Antiviral Mechanisms That Can Be Exploited for the Development of mRNA-Mediated Gene Expression Strategy. Stem Cells Dev..

[74] Guo Y. (2019). The Underdeveloped Innate Immunity in Embryonic Stem Cells: The Molecular Basis and Biological Perspectives from Early Embryogenesis. Am. J. Reprod. Immunol..

[75] D’Angelo W., Acharya D., Wang R., Wang J., Gurung C., Chen B., Bai F., Guo Y.-L. (2016). Development of Antiviral Innate Immunity During In Vitro Differentiation of Mouse Embryonic Stem Cells. Stem Cells Dev..

[76] Romeike M., Spach S., Huber M., Feng S., Vainorius G., Elling U., Versteeg G.A., Buecker C. (2022). Transient Upregulation of IRF1 during Exit from Naive Pluripotency Confers Viral Protection. EMBO Rep..

[77] Eggenberger J., Blanco-Melo D., Panis M., Brennand K.J., tenOever B.R. (2019). Type I Interferon Response Impairs Differentiation Potential of Pluripotent Stem Cells. Proc. Natl. Acad. Sci. USA.

[78] Gurung C., Fendereski M., Sapkota K., Guo J., Huang F., Guo Y.-L. (2021). Dicer Represses the Interferon Response and the Double-Stranded RNA-Activated Protein Kinase Pathway in Mouse Embryonic Stem Cells. J. Biol. Chem..

[79] Sansom S.N., Griffiths D.S., Faedo A., Kleinjan D.-J., Ruan Y., Smith J., Van Heyningen V., Rubenstein J.L., Livesey F.J. (2009). The Level of the Transcription Factor Pax6 Is Essential for Controlling the Balance between Neural Stem Cell Self-Renewal and Neurogenesis. PLoS Genet..

[80] Roy E., Cao W. (2022). Glial Interference: Impact of Type I Interferon in Neurodegenerative Diseases. Mol. Neurodegener..

[81] Tan P.-H., Ji J., Hsing C.-H., Tan R., Ji R.-R. (2022). Emerging Roles of Type-I Interferons in Neuroinflammation, Neurological Diseases, and Long-Haul COVID. Int. J. Mol. Sci..

[82] Jin M., Xu R., Wang L., Alam M.M., Ma Z., Zhu S., Martini A.C., Jadali A., Bernabucci M., Xie P. (2022). Type-I-Interferon Signaling Drives Microglial Dysfunction and Senescence in Human iPSC Models of Down Syndrome and Alzheimer’s Disease. Cell Stem Cell.

[83] Viengkhou B., Hofer M.J. (2023). Breaking down the Cellular Responses to Type I Interferon Neurotoxicity in the Brain. Front. Immunol..

[84] Ben-Yehuda H., Matcovitch-Natan O., Kertser A., Spinrad A., Prinz M., Amit I., Schwartz M. (2020). Maternal Type-I Interferon Signaling Adversely Affects the Microglia and the Behavior of the Offspring Accompanied by Increased Sensitivity to Stress. Mol. Psychiatry.

[85] Pavlinek A., Matuleviciute R., Sichlinger L., Dutan Polit L., Armeniakos N., Vernon A.C., Srivastava D.P. (2022). Interferon-γ Exposure of Human iPSC-Derived Neurons Alters Major Histocompatibility Complex I and Synapsin Protein Expression. Front. Psychiatry.

[86] Simeone A. (1998). Otx1 and Otx2 in the Development and Evolution of the Mammalian Brain. EMBO J..

[87] Beby F., Lamonerie T. (2013). The Homeobox Gene Otx2 in Development and Disease. Exp. Eye Res..

[88] Su Z., Zhang Y., Liao B., Zhong X., Chen X., Wang H., Guo Y., Shan Y., Wang L., Pan G. (2018). Antagonism between the Transcription Factors NANOG and OTX2 Specifies Rostral or Caudal Cell Fate during Neural Patterning Transition. J. Biol. Chem..

[89] Samara A., Spildrejorde M., Sharma A., Falck M., Leithaug M., Modafferi S., Bjørnstad P.M., Acharya G., Gervin K., Lyle R. (2022). A Multi-Omics Approach to Visualize Early Neuronal Differentiation in 4D. bioRxiv.

[90] Kuang Y.-L., Munoz A., Nalula G., Santostefano K.E., Sanghez V., Sanchez G., Terada N., Mattis A.N., Iacovino M., Iribarren C. (2019). Evaluation of Commonly Used Ectoderm Markers in iPSC Trilineage Differentiation. Stem Cell Res..

[91] Suter D.M., Tirefort D., Julien S., Krause K.-H. (2009). A Sox1 to Pax6 Switch Drives Neuroectoderm to Radial Glia Progression during Differentiation of Mouse Embryonic Stem Cells. Stem Cells.

[92] Venere M., Han Y.-G., Bell R., Song J.S., Alvarez-Buylla A., Blelloch R. (2012). Sox1 Marks an Activated Neural Stem/Progenitor Cell in the Hippocampus. Development.

[93] Liu X., Fang Z., Wen J., Tang F., Liao B., Jing N., Lai D., Jin Y. (2020). SOX1 Is Required for the Specification of Rostral Hindbrain Neural Progenitor Cells from Human Embryonic Stem Cells. Iscience.

[94] Ustyantseva E.I., Medvedev S.P., Vetchinova A.S., Minina J.M., Illarioshkin S.N., Zakian S.M. (2019). A Platform for Studying Neurodegeneration Mechanisms Using Genetically Encoded Biosensors. Biochem. Mosc..

[95] Grigor’eva E.V., Malankhanova T.B., Surumbayeva A., Pavlova S.V., Minina J.M., Kizilova E.A., Suldina L.A., Morozova K.N., Kiseleva E., Sorokoumov E.D. (2020). Generation of GABAergic Striatal Neurons by a Novel iPSC Differentiation Protocol Enabling Scalability and Cryopreservation of Progenitor Cells. Cytotechnology.

[96] Livak K.J., Schmittgen T.D. (2001). Analysis of Relative Gene Expression Data Using Real-Time Quantitative PCR and the 2- ΔΔCT Method. Methods.

