# Supplementary material for: The Generation of Genetically Engineered Human Induced Pluripotent Stem Cells Overexpressing IFN-β for Future Experimental and Clinically Oriented Studies
Source: Int J Mol Sci. 2024 Nov 20;25(22):12456. doi: 10.3390/ijms252212456 (PMC11595023; doi:10.3390/ijms252212456)
Supplement: Supplementary file 1 [file ijms-25-12456-s001.zip › Supplement_Figure_S1,S2,S3,S4,S5.pdf]

SUPPLEMENTAL DATA

The generation of genetically engineered human induced pluripotent stem cells overexpressing IFN-β for future experimental and clinically oriented studies

Olga Sheveleva[1*], Elena Protasova[1], Elena Grigor'eva[2], Nina Butorina[1], Valeriia Kuziaeva[1], Daniil Antonov[1], Victoria Melnikova[3], Sergey Medvedev[2], Irina Lyadova[1*]

[1] Laboratory of Cellular and Molecular Basis of Histogenesis, Koltzov Institute of Developmental Biology of the Russian Academy of Sciences, Moscow, 119334, Russia

[2] Laboratory of Developmental Epigenetics, Institute of Cytology and Genetics, Siberian Branch of Russian Academy of Sciences, Novosibirsk, 630090, Russia

[3] Laboratory of Comparative Developmental Physiology, Koltzov Institute of Developmental Biology of the Russian Academy of Sciences, Moscow, 119334, Russia

* Correspondence: ivlyadova@mail.ru; Tel.: +7 499-135-87-80

* Correspondence: on_sheveleva@mail.ru; Tel.: +7 499-135-87-80

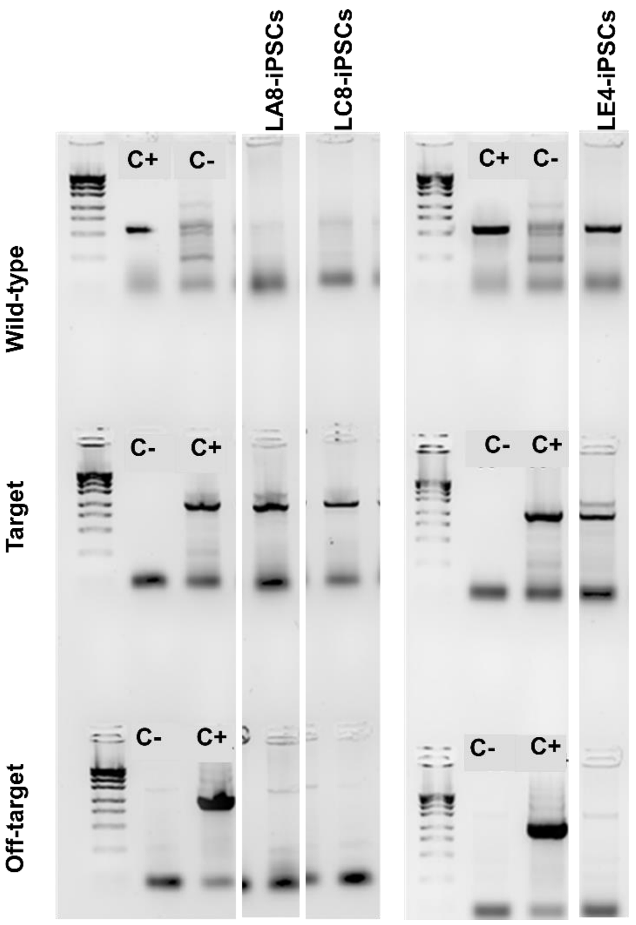

Supplementary Figure S1. Gels showing the results of IFN-iPSC (line LC8, LA8, LE4) screening for on-target and off-target inserts. **Wild type *AAVS* loci (WT *AAVS1*)**, C+, positive control, parental K7-iPSCs containing unmodified *AAVS1* loci; C-, negative control, a previously generated iPSC line with a proven correct insert of roGFP2-Orp1 transgene into *AAVS1* locus; **target** - screening in edited iPSCs, C+, iPSC line with a proven correct insert of roGFP2-Orp1 transgene into *AAVS1* locus, C-, parental K7-iPSC line; **off-target** -screening in edited iPSCs, C+, pAAVS1-hPGK-IFNB1 plasmid; C-, iPSC line with a proven correct insert into the *AAVS1* locus. iPSCs, induced pluripotent stem cells; K7-iPSCs, parental K7-4Lf iPSC line.

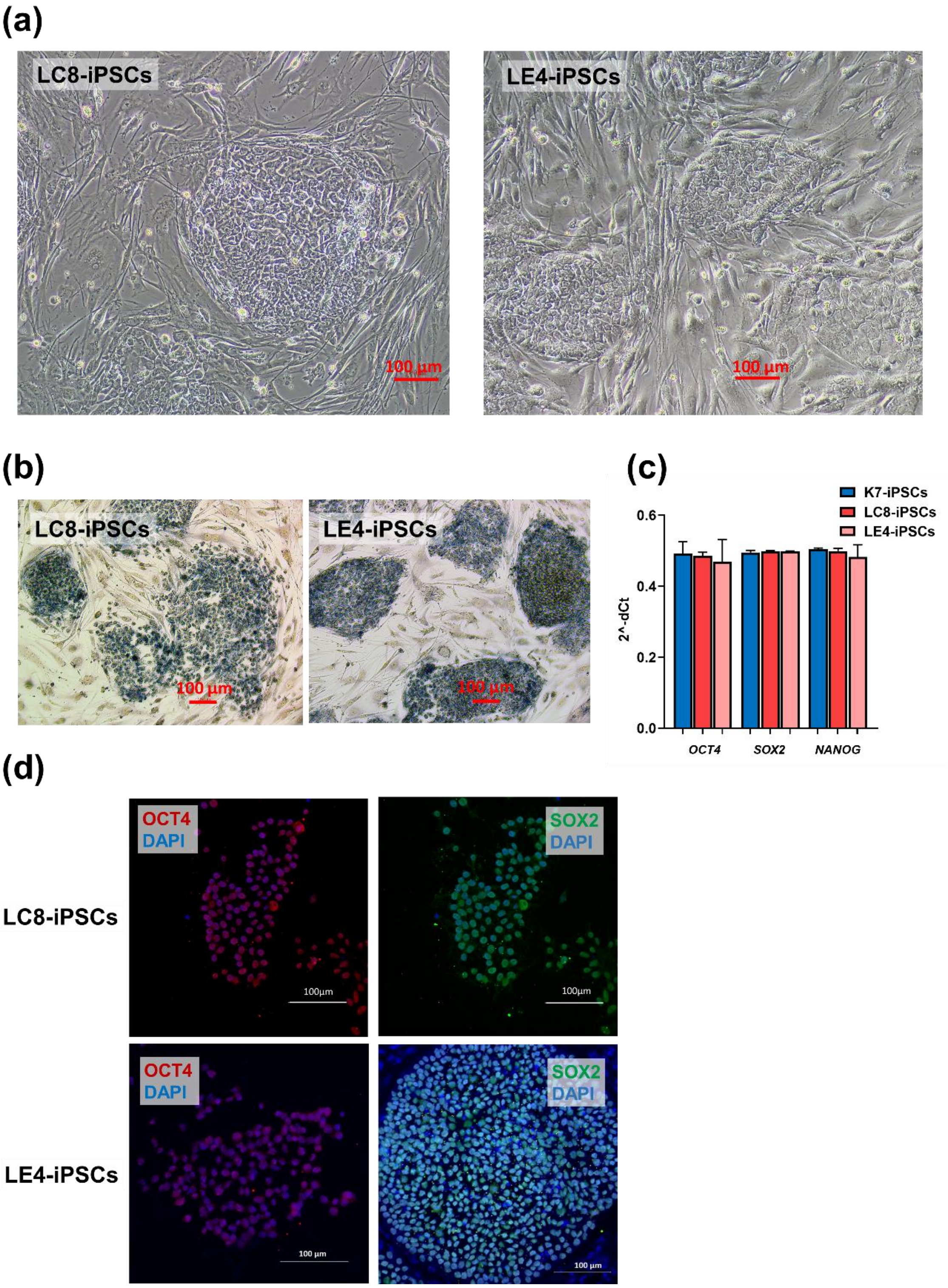

Supplementary Figure S2. IFNB-iPSC lines display the morphological and phenotypic characteristics of pluripotent cells. The results obtained using IFNB-iPSC line LA8 and LE4 are shown. **(a)** Light microscopy of LC8-iPSCs and LE4-iPSCs growing on mouse embryonic fibroblast feeder cells. Phase contrast. Magnification, 10x. **(b)** Positive immunohistochemical staining of LC8-iPSCs and LE4-iPSCs for alkaline phosphatase**. (c)** The expression of pluripotency markers *OCT4*, *SOX2* and *NANOG* by LC8-iPSCs and LE4-iPSCs. **(d)** The expression of OCT4 (red) and SOX2 (green) pluripotency proteins in LC8-iPSCs and LE4-iPSCs. Immunofluorescence staining, confocal microscopy (Zeiss LSM 880 microscope; Carl Zeiss, Jena, Germany). Nuclei were stained with DAPI (blue). The scale bar is 50 µm or 100 µm.

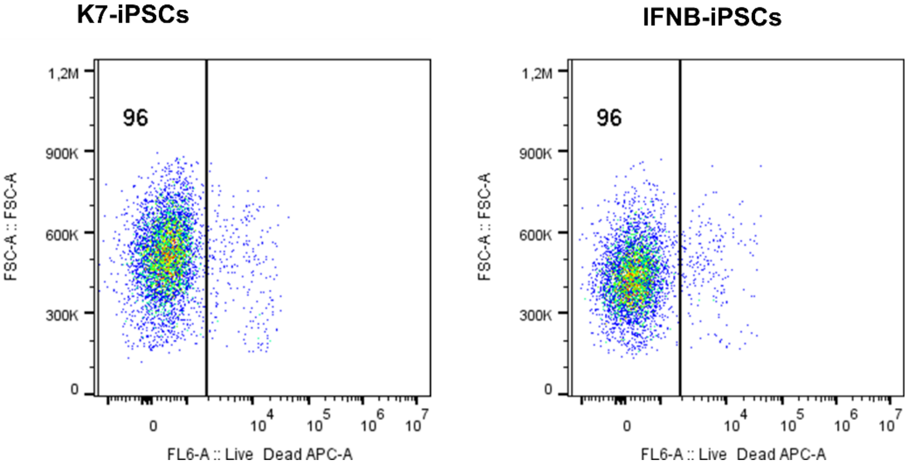

Supplementary Figure S3. Overexpression of IFNB does not affect the viability of iPSC cells. IFNB-iPSCs and K7-iPSCs were simultaneously treated with TrypLE Express Enzyme, washed and stained using the LIVE/DEAD™ Fixable Far Red Dead Cell Stain Kit. The percentages of live cells were determined after sequentially gating on large (FSC-A versus SSC-A) and singlet (FSC-A versus FSC-H) cells.

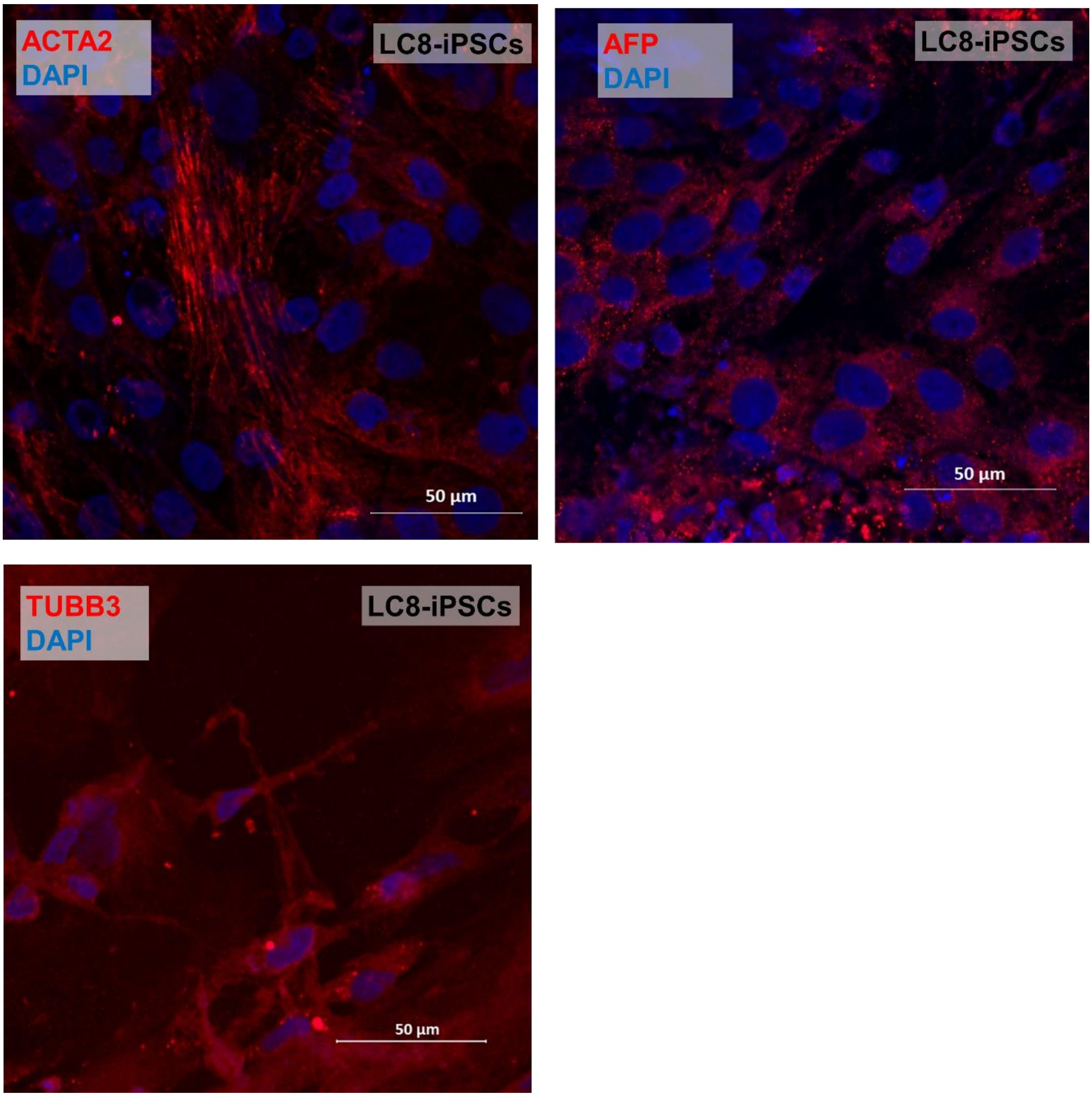

Supplementary Figure S4. Immunofluorescence analysis reveals the expression of endoderm-, mesoderm- and ectoderm-associated proteins in embryonic bodies spontaneously differentiated from LC8-iPSCs. Cells were stained with antibodies specific to endoderm (AFP), mesoderm (ACTA2) and ectoderm markers (TUBB3) and analyzed by confocal microscopy (Zeiss LSM 880 (Carl Zeiss, Jena, Germany). Nuclei were stained with DAPI (blue). The scale bar: 50 µm.

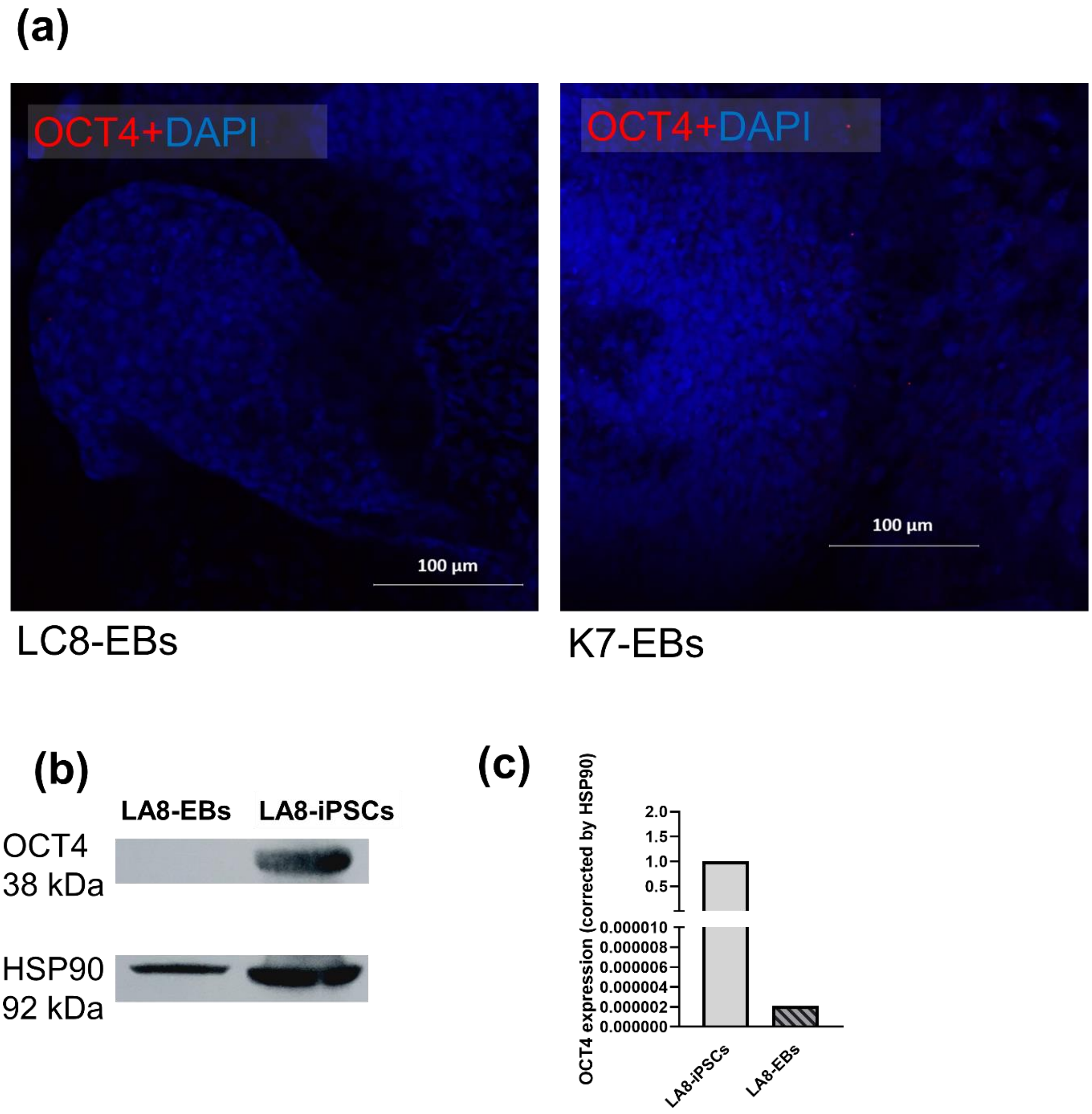

Supplementary Figure S5. Examination the pluripotency of IFNB- and K7-EBs. (**a**) The expression of OCT4 (red) - pluripotency protein in LC8-EBs and K7-EBs (21 day). Immuno-fluorescence staining, confocal microscopy (Zeiss LSM 880 microscope; Carl Zeiss, Jena, Germany). Nuclei were stained with DAPI (blue). (**b**) Western blot analysis of OCT4 protein in LA8-iPSCs and LA8-EBs. (**c**) Figure indicates fold-change of OCT4 expression in LA8-EBs relative to LA8-iPSCs (calculated based on the densitometric analysis of OCT4 and HSP90 expressions)
